# Construction of Personalized Predictive Models for Missed Medication Doses Using Wearable Device Data: Prospective Observational Study

**DOI:** 10.2196/72113

**Published:** 2025-06-24

**Authors:** Haru Iino, Hayato Kizaki, Shungo Imai, Satoko Hori

**Affiliations:** 1 Division of Drug Informatics, Faculty of Pharmacy and Graduate School of Pharmaceutical Sciences, Keio University, 1-5-30 Shibakoen, Minato-ku, Tokyo, 105-8512, Japan, 81 3-5400-2650

**Keywords:** personalized predictive models, missed medication doses, wearable device data, Light Gradient Boosting Machine, LightGBM

## Abstract

**Background:**

Declining medication adherence remains a critical health care issue, often assessed through unreliable self-reporting methods. Wearable devices (WDs) may offer an objective means to improve adherence monitoring by continuously recording physiological and activity data.

**Objective:**

This study aimed to develop and internally validate personalized predictive models, utilizing objective physiological and activity data from WDs, for identifying missed medication doses.

**Methods:**

A 30-day prospective observational study was conducted with 8 participants who wore Apple Watches and used a dedicated iOS app. The app collected demographics, medication details, psychological factors, mealtimes, and daily missed dose events. WDs recorded time-series data (ie, activity, heart rate, sleep) at 3-minute intervals. Data were aggregated into 1-hour segments, and lag (6 and 12 h) as well as rolling (24 h) features were generated. Light Gradient Boosting Machine models were constructed for each individual’s dosing regimen if the missed dose rate exceeded 20%. Two modeling approaches were compared: a group cross-validation (CV) model that grouped data by day to avoid data leakage from rolling features, and a nonrolling feature model that excluded rolling features and used leave-one-out CV. *F*_1_-score, accuracy, recall, and precision were assessed between the 2 models.

**Results:**

Of the 15 enrolled participants, 8 completed the study; 4 had a missed dose rate above 20%. In these 4 individuals, the group CV model achieved *F*_1_-scores of 0.435 to 0.902, with accuracy ranging from 0.711 to 0.911, recall from 0.278 to 0.822, and a precision of 1.000 for the most robust regimens. The nonrolling feature model yielded *F*_1_-scores of 0.667 to 0.910, with accuracy ranging from 0.800 to 0.906, recall from 0.500 to 0.835, and a precision of 1.000. Morning dosing regimens generally showed higher predictive performance than evening or afternoon. Time-series features, particularly those reflecting 6-, 12-, and 24-hour patterns, emerged as key predictors, indicating that physiological and lifestyle variations prior to dosing strongly influenced missed dose events.

**Conclusions:**

Personalized predictive models using WD-derived data demonstrated high precision for detecting missed medication doses, especially in morning and evening regimens. These findings underscore the feasibility of employing continuous, objective physiological and activity data from WDs to forecast nonadherence events. Although the sample size was limited, restricting the generalizability of the results, this study demonstrates the potential of WD-based personalized prediction of medication adherence. Future work should involve larger populations for external validation, strategies to improve recall, especially for clinically critical medications, and careful consideration of real-world implementation challenges.

## Introduction

Medication adherence is a crucial factor in pharmaceutical care [1]. A 2020 study revealed that 32% of patients did not take their medications correctly, and those who did not adhere properly incurred an additional average monthly cost of US $97.98 [2]. This indicates that medication nonadherence imposes both health and economic burdens. Given these significant impacts, identifying effective strategies to improve medication adherence has become a critical health care priority. To prevent a decline in medication adherence, it is necessary to accurately assess adherence at an individual level [3,4]. Typically, medication adherence is evaluated by comparing the frequency of clinic visits with prescription intervals or by simple questioning [5-7]. However, this approach faces challenges such as time constraints and the reliance on self-reporting [5], which is often subject to recall bias and social desirability bias, limiting its reliability for accurate monitoring [8].

Various factors influence medication adherence and are closely related to the patient’s lifestyle [9-12]. We previously conducted a study on the relative importance of factors related to medication adherence and suggested that the consistency of lifestyle habits may be the most important factor. Some questionnaires that have been used to evaluate medication adherence include questions about the patient’s lifestyle [5,13-16], as inquiring about an individual’s lifestyle is a common first step toward improving adherence [12]. Lifestyle inquiries often relate to the number of meals consumed and include subjective lifestyle evaluations. However, it is currently difficult to quantitatively evaluate comprehensive aspects of lifestyle beyond meal frequency using objective indicators [17-19].

Therefore, we focused on wearable devices (WDs) to continuously monitor a wearer’s physiological and activity data. WDs accumulate daily information, such as the wearer’s activity level, heart rate, and sleep duration, allowing the collection of objective data [20-23], which may reflect a patient’s lifestyle [24]. Thus, by constructing machine learning models from these data, it may be possible to determine predictive factors related to a patient’s medication adherence. We previously conducted a scoping review on medication adherence management using WDs, which revealed that, while reminders and alerts have been widely used in medication management, some research methods use WD data as an advanced approach to medication adherence management [25]. Leveraging WD data, which have not been widely used to date, and obtaining insights into medication adherence from unconventional objective information may result in new medication management approaches that place less burden on both patients and health care providers. Recent studies have demonstrated that data-driven approaches using deep learning and ensemble methods significantly enhance prediction accuracy of medication adherence compared to traditional self-reporting methods [26]. Additionally, WDs have shown promise in improving clinical decision-making through real-time, noninvasive physiological data collection, particularly in the management of chronic diseases such as heart failure [27]. However, successful long-term monitoring using WDs in clinical practice requires consideration of usability and acceptability, especially among older adults, as well as strategies to maintain continuous user engagement and address technical challenges [28]. Moreover, while machine learning approaches for adherence prediction are advancing, studies also indicate that many existing predictive models have a high risk of bias, highlighting the need for enhanced methodological quality [29].

Therefore, addressing the gaps and the need for objective, personalized adherence monitoring, the primary aim of this study was to develop and internally validate personalized predictive models for identifying missed medication doses, utilizing objective physiological and activity data prospectively collected from commonly used WDs.

## Methods

### Development of Data Collection Platform and Patient Recruitment

**Figure 1.**
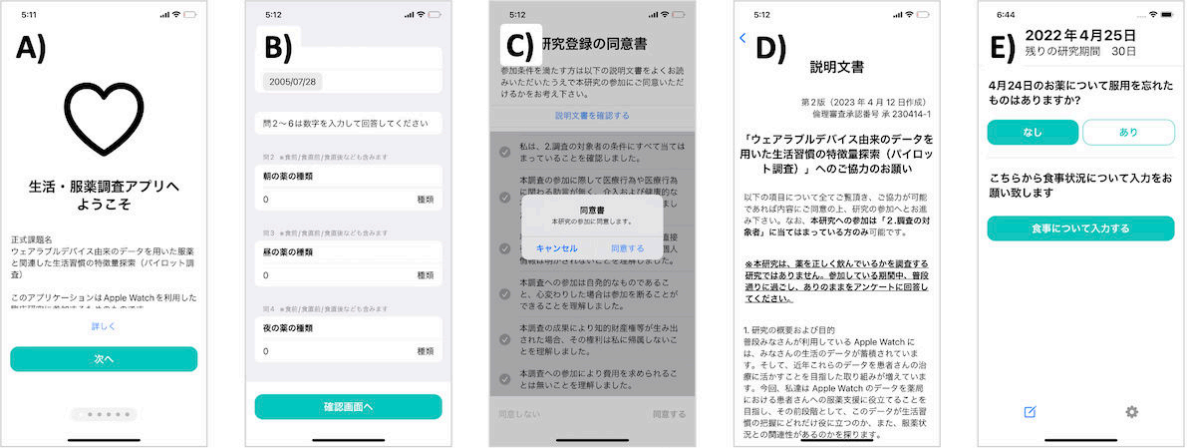
Screenshots of the dedicated study app, including (A) the launch screen, (B) initial questionnaire screen, (C) consent form and agreement screen, (D) research explanation document, and (E) regular survey screen (input on medication status).

Participants were recruited between May 2023 and December 2023. All study recruitment and procedures were conducted in Japan. Information about the study was disseminated through flyers posted in local libraries and via a dedicated project page hosted on our laboratory’s website, inviting interested individuals to voluntarily download the dedicated study app. Eligibility criteria for participation, which were confirmed via an initial in-app questionnaire after its download and prior to obtaining informed consent, were as follows: (1) aged 18 years or older; (2) taking at least 1 medication prescribed by a hospital with instructions for daily use for an intended duration of at least 3 months (individuals whose only medications were for self-adjustment, eg, pro re nata or “as needed” only, were excluded); (3) routinely using an Apple Watch (defined as wearing it for an estimated minimum of 8 hours per day, at least 5 days a week) and an iPhone; and (4) able to download the dedicated study app from the Japanese App Store onto their iPhone and operate the app. The app was developed specifically for iOS smartphones and made available exclusively on the Japanese App Store (Figure 1). It included a questionnaire and health data–sharing capabilities, with collected responses and health data stored on a cloud server.

The questionnaires collected information on age, usage, types of medications taken, psychological factors related to adherence (10 questions on a 5-point scale), nonworking days of the week, meal start times and durations, and the presence or absence of missed doses over 30 days. The WD data collected included exercise time, standing time, active energy, resting energy expenditure, walking and running distances, step count, number of flights climbed, maximum oxygen uptake, mindfulness time, vital signs (ie, heart rate, heart rate variability, resting heart rate), oxygen saturation levels, and sleep time. Specifically, health data, excluding those related to the questionnaires and sleep (such as wake-up and bedtime), were obtained as time-series data divided into 3-minute intervals. Missing values arising from nonwear periods or lack of body movement were imputed with zeros. This simple imputation approach was chosen for its feasibility in this initial exploratory study. While zero values might reasonably represent periods of inactivity for some metrics like step counts, we acknowledge this method is suboptimal for physiological measures (eg, heart rate) and that the assumption that missingness equates to zero may introduce bias if nonwear periods systematically correlate with adherence behaviors. Alternative imputation strategies (eg, mean and median imputation, interpolation, forward and backward fill) exist, and investigating their impact on model performance represents an area for future refinement. The subsequent aggregation of the 3-minute data into 1-hour descriptive statistics may, however, partially mitigate the direct influence of the initial imputation choice on the final model features.

The participants downloaded the app, reviewed the research explanation materials and consent form within the app, and joined the study after providing informed consent. After initiating the survey, an initial questionnaire screen was displayed to collect information on age, medication usage, and medication types. Subsequently, the app transitioned to a regular survey screen, where the participants recorded their medication status up to the previous day at their convenience.

### Preprocessing Before Model Construction and Verification of Time-Series Components

To evaluate the quality of the collected time-series data from WDs, the completeness of each data type was visualized. Insufficiently recorded data were excluded from further analyses (Multimedia Appendix 1). The periodicity of the included time-series components was examined using additive decomposition, confirming the absence of trend components and the presence of seasonal variations on a daily basis (Multimedia Appendix 1). Spectral analysis with fast Fourier transformation was performed to verify the lengths of the periods, confirming that many data types exhibited strong spectral peaks at 24- or 12-hour intervals (Multimedia Appendix 1).

To construct predictive models, it is desirable to ensure a sufficient number of event occurrences and to avoid extreme imbalances in the distribution of response variables. Therefore, the missed dose events were aggregated. Using the number of events during the observation period, the event occurrence rate was calculated using the following equation: missed dose event occurrence rate=(number of missed dose events)/(number of doses that should have been taken during the period).

In this study, medication adherence refers to the value calculated as above.

### Confirmation of Response Variables and Creation of Time-Series Features

All data were divided into 1-hour intervals. For each time segment, descriptive statistics, including the mean, variance, maximum, minimum, median, and sum, were calculated. Assuming that the state prior to each time point is related to the event, time-series–related features, such as lag features for the previous 6 and 12 hours, and rolling features for the previous 24 hours, were added. The data were classified by individual and dosing regimens (morning, afternoon, and evening). For the portions where a sufficient number of events (>20%) were secured, models to detect missed medication events from the WD data were created.

In terms of the dosing regimen, specific timeslots where events could potentially occur were allocated: 6 AM to 10:59 AM for morning doses, 11 AM to 2:59 PM for afternoon doses, and 6 PM to 11:59 PM for evening doses. For example, data from 6 PM to 11:59 PM were extracted when constructing a model to predict evening events, resulting in 6 datasets divided into 1-hour intervals per day. If an event occurred on that day, it was labeled as 1; otherwise, it was labeled as 0 as the response variable. If the data were recorded for 30 days, 180 datasets (6×30) were used for model construction.

### Construction of Predictive Models

Predictive models were constructed using Light Gradient Boosting Machine (LightGBM). LightGBM was selected due to its demonstrated high predictive performance on tabular data tasks similar to ours, its computational efficiency in terms of training speed and memory usage compared to other gradient boosting algorithms, and its built-in capability to provide feature importance rankings, which was utilized in our analysis. Given that the data were divided into 1-hour intervals, the newly created rolling features were generated based on the past 24 hours. In other words, for adjacent time points, only 1 hour of data differed while the remaining 23 hours of data were the same, potentially resulting in minimal changes. Therefore, performing random cross-validation (CV) can lead to other rolling features from the same day being learned and used as test data during prediction, which may not be appropriate.

To prevent this, the following two types of models were constructed [30]: (1) a model in which data from the same day were grouped together and group CV was performed (group CV model) and (2) a model in which rolling features were excluded from the variables and leave-one-out CV was performed (nonrolling feature model). The models were constructed to optimize the *F*_1_-scores. Evaluation metrics, such as the *F*_1_-score, accuracy, recall, and precision, were calculated [31]. In addition, the feature importance of each model was computed for each fold in the CV, and the average for each model was provided. All analyses were performed using Python 3.10.12, and the code used in prediction models is present in Multimedia Appendix 2.

### Ethical Considerations

This study complied with the Ethical Guidelines for Medical and Biological Research Involving Human Subjects published by the Japanese Government (Ministry of Health, Labour and Welfare; Ministry of Education, Culture, Sports, Science and Technology; and Ministry of Economy, Trade and Industry). All research plans were reviewed and approved by the Research Ethics Committee of Keio University Faculty of Pharmacy (23414‐1). We completed a checklist for reporting studies involving machine learning, following recommended guidelines for such research (Checklist 1) [32].

Informed consent was obtained electronically from all participants via the dedicated study app before they commenced any study procedures. The consent form provided detailed information regarding the study’s objectives, procedures (including the collection of questionnaire data and continuous physiological and activity data from their Apple Watch), potential risks and benefits, data handling, and the voluntary nature of participation. Participation was entirely voluntary, and participants were clearly informed of their right to withdraw from the study at any time, for any reason, without prejudice or penalty, fully ensuring patient autonomy. To protect privacy and confidentiality, all data collected through the app and wearable device were anonymized at the point of collection before being transmitted and stored. Anonymized data were managed on a secure cloud server, employing appropriate technical and administrative safeguards to prevent unauthorized access, disclosure, or loss. Although this study was conducted exclusively in Japan using a locally distributed app, and thus not directly subject to General Data Protection Regulation or Health Insurance Portability and Accountability Act regulations, data management practices were designed to align with principles of robust data protection and security suitable for sensitive health-related information. The participants who completed the 30-day survey were compensated with Japanese ¥3000 (US $20.84).

## Results

### Aggregation of Event Occurrence Rates

Fifteen individuals participated in the survey, and 8 completed it. A summary of the participants’ data is shown in Table 1. Among them, 4 (50%) were aged 20-29 years, 2 (25%) were aged 40-49 years, 1 (12.5%) was aged 50-59 years, and 1 (12.5%) did not provide an answer regarding age. The collected data were divided into individual and dosing regimens, resulting in 15 arms. Among these arms, there were 4 in which the event occurrence rate was >20% (ID1: morning, evening; ID2: evening; ID3: afternoon). These event occurrence rates ranged from 41.4% to 56.7%, without significant skewness, and were used as response variables for predictive model construction. For individuals ID4 and ID5, although a few events were observed in the evening, none were observed in the morning. For individuals ID6, ID7, and ID8, no events were observed. The event occurrence rate per number of days, without distinguishing the dosing regimens for each individual, are shown in Table S1 in Multimedia Appendix 3, and the event occurrence rates per total number of doses are provided in Table S2 in Multimedia Appendix 3.

**Table 1.** Summary of the number of events and medication adherence rates for 8 adult Apple Watch users in Japan from a 30-day prospective observational study monitoring daily medication use.

ID (age group) and administration		Prescribed medications, n	Expected doses, n	Events occurring, n	Missed dose event occurrence rate, %
ID1 (20-29 years)					
	Morning (after a meal)	1	30	17	56.7
	Evening (after a meal or before bed)	3	30	15	50
ID2 (20-29 years)					
	Evening (after a meal)	1	30	17	56.7
ID3 (20-29 years)					
	Morning (before and after a meal)	6	29	2	6.9
	Afternoon (after a meal)	1	29	12	41.4
	Evening (after a meal)	9	29	1	3.4
ID4 (20-29 years)					
	Morning (after a meal)	5	30	0	0
	Evening (before bed)	1	30	4	13.3
ID5 (40-49 years)					
	Morning (after a meal)	1	29	0	0
	Evening (after a meal)	2	29	1	3.4
ID6 (50-59 years)					
	Morning (after a meal)	4	30	0	0
	Evening (after a meal)	3	30	0	0
ID7 (no answer)					
	Morning (after a meal)	4	30	0	0
	Evening (after a meal or before bed)	4	30	0	0
ID8 (50-59 years)					
	Morning (after a meal)	2	30	0	0

### Predictive Models

The results of the group CV model are presented in Table 2, and those of the nonrolling feature model are presented in Table 3. Internal validation showed that the *F*_1_-score ranged from 0.435 to 0.902, accuracy from 0.711 to 0.911, and recall from 0.278 to 0.822. In the nonrolling feature model, the *F*_1_-score ranged from 0.667 to 0.910, accuracy from 0.800 to 0.906, and recall from 0.500 to 0.835. Precision was 1.0 for both models.

**Table 2.** Performance of Light Gradient Boosting Machine group cross-validation models in predicting missed medication doses for 4 participants (adult Apple Watch users, Japan) from a 30-day prospective observational study.

		*F*_1_-score	Accuracy	Recall	Precision
ID1					
	Morning (after a meal)	0.902	0.911	0.822	1.000
	Evening (after a meal or before bed)	0.784	0.807	0.659	1.000
ID2					
	Evening (after a meal)	0.793	0.806	0.657	1.000
ID3					
	Afternoon (after a meal)	0.435	0.711	0.278	1.000

**Table 3.** Performance of Light Gradient Boosting Machine nonrolling feature models (leave-one-out cross-validation) in predicting missed medication doses for 4 participants (adult Apple Watch users, Japan) from a 30-day prospective observational study.

		*F*_1_-score	Accuracy	Recall	Precision
ID1					
	Morning (after a meal)	0.910	0.906	0.835	1.000
	Evening (after a meal or before bed)	0.816	0.844	0.689	1.000
ID2					
	Evening (after a meal)	0.867	0.867	0.765	1.000
ID3					
	Afternoon (after a meal)	0.667	0.800	0.500	1.000

### Feature Importance

**Figure 2.**
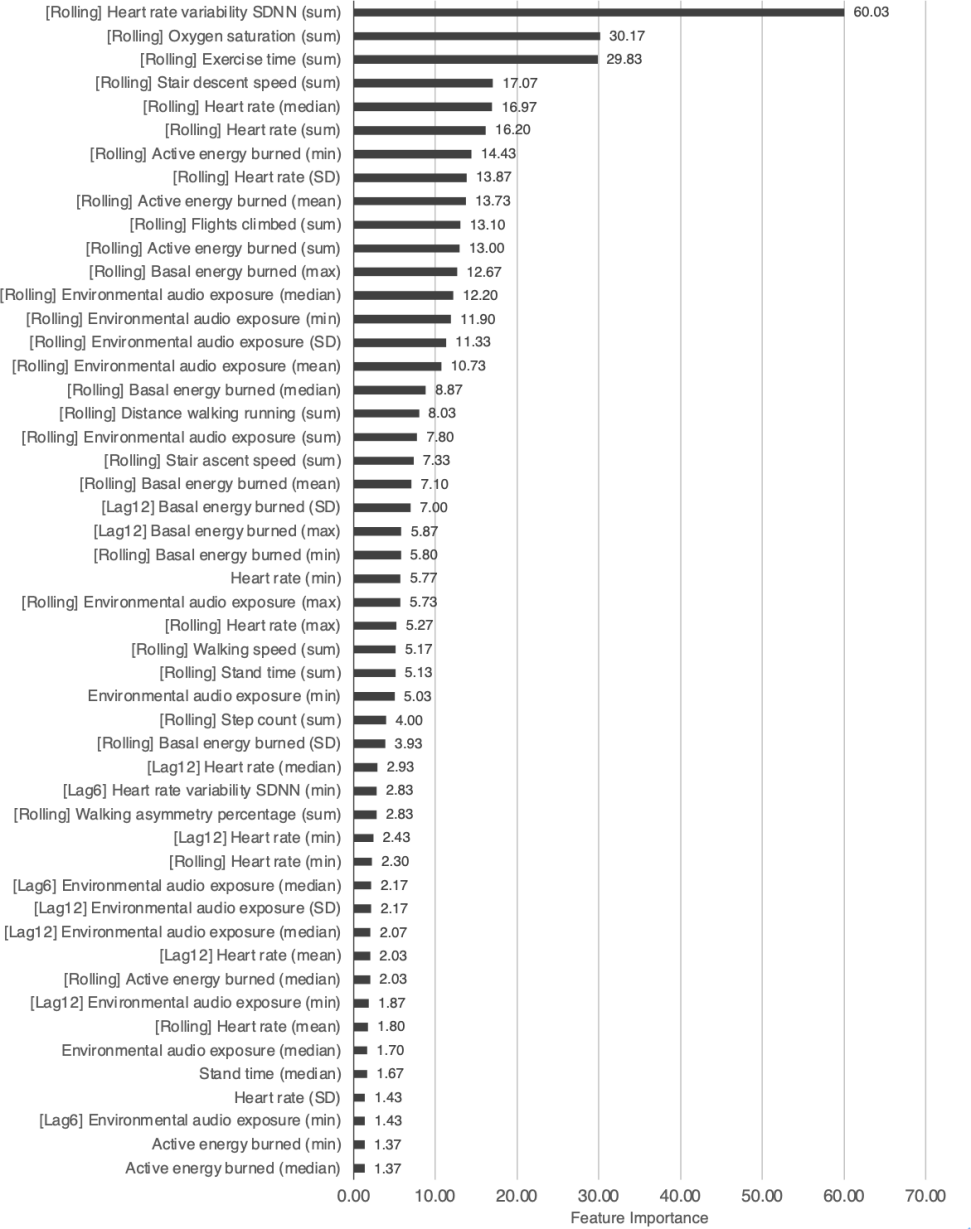
Top 50 feature importance in the group cross-validation model for ID1: morning (after a meal). SDNN: standard deviation of all normal to normal R-R (NN) intervals; [Lag6]: 6-hour lag features; [Lag12]: 12-hour lag features; [Rolling]: rolling feature.

**Figure 3.**
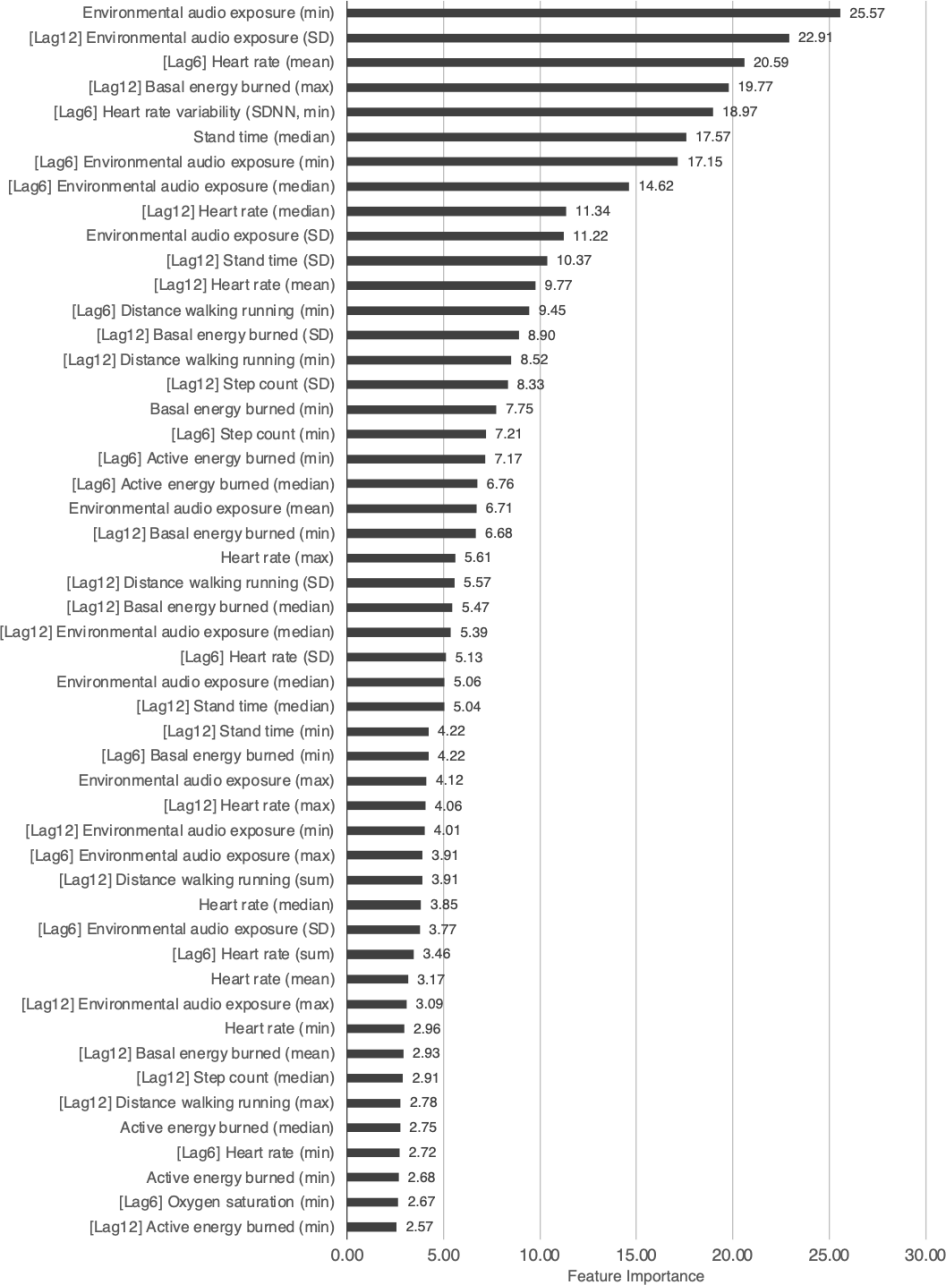
Top 50 feature importance in the nonrolling feature model for ID1: morning (after a meal). SDNN: standard deviation of all normal to normal R-R (NN) intervals; [Lag6]: 6-hour lag features; [Lag12]: 12-hour lag features; [Rolling]: rolling feature.

The importance of the top 50 features in the group CV model for ID1 is shown in Figure 2, and the importance of the top 50 features in the nonrolling feature model is shown in Figure 3. The feature importance values of the other models are present in Multimedia Appendix 4 .

## Discussion

### Findings

In this prospective observational study, we collected WD data and developed 2 models using missed medication events as response variables for each individual and dosing regimen. The internal validation demonstrated good overall performance, except for the afternoon dosing model. For morning and evening dosing regimens, the maximum *F*_1_-score, accuracy, and recall were 0.902, 0.911, and 0.822, respectively, with precision consistently reaching 1.0, indicating a high discriminative performance. The performance ranking by regimen was morning > evening > afternoon, and the 2 types of models showed generally equivalent results. In terms of feature importance, time-series features consistently ranked as highly important across models.

A key contribution of this study was the development of an integrated system for collecting medication information and WD data to construct predictive models. We herein performed app development, database construction, and implementation of a prospective data collection protocol involving study participants. Despite the limited sample size, this is the first study to build a predictive model for missed medication doses using activity, heart rate, and other physiological data obtained from commonly used WDs, targeting the general patient population. This methodological approach, focusing on personalized prediction using objective, continuously monitored data streams, represents the core novelty of the study. By addressing the challenges of real-world data collection and focusing on commonly used devices, this study could serve as a novel foundation for advancing medication adherence monitoring.

### High Precision and Low Recall in the Models

Both the group CV and nonrolling feature models achieved high precision, representing their ability to accurately identify missed medication events with minimal false positives. However, the recall was relatively low, indicating that some missed events were not detected. This limitation was particularly evident during the daytime and evening regimens, likely due to the large variations in medication patterns and lifestyle habits during these periods, which the models have not sufficiently learned. Additionally, certain missed events may not generate measurable changes in WD data, such as forgetting to take medications without altering measurable activity levels or physiological parameters.

In this study, the models were optimized for *F*_1_-scores to balance precision and recall. Consequently, the precision was high, and recall was low. In situations where adherence is particularly critical, such as with immunosuppressant or anticoagulant medications, it is necessary to detect missed doses more accurately. When targeting such medications, it may be necessary to construct models emphasizing recall, such as cost-sensitive learning [33].

### Factors Influencing Afternoon Model Performance

Two primary factors might explain the poorer performance of the afternoon models. First, unique influences on afternoon medication adherence compared with those on morning or evening medication adherence. During the day, activities, such as going out, work, and school, and factors such as inaccessibility to medication or the impact of work and eating out can influence adherence [34-36]. When patients do not have their medication on their person, despite no significant lifestyle changes, WDs may not sufficiently capture such situations, potentially reducing the predictive model’s performance. Second, the shorter time window allocated to the afternoon (4 h) compared to the morning (5 h) and evening (6 h) may have limited data volume, possibly preventing the model from utilizing sufficient data for learning. However, the evening model, despite possessing more data, did not outperform the morning model. This could be because there is generally more variation in activities during the evening than during the morning [37,38], making evening predictions more challenging.

### Feature Importance in the 2 Models

Feature importance analysis highlighted the significance of time-series features. In the group CV model, the rolling features ranked high, suggesting that data obtained within the previous 24 hours significantly influenced the prediction of missed medications [39]. Similarly, in the nonrolling feature model, the lag features (from 6 and 12 hours prior) were among the most important. The fact that these 2 time-series features rank higher than other features at the time of the event implies that physiological and lifestyle changes preceding missed doses are greater predictors of a missed dose compared with the immediate conditions at the time of the event.

Although both models demonstrated similar overall performance, the nonrolling feature model showed slight superiority over the group CV model. The nonrolling feature model utilized 270 input variables, which is a relatively large number compared to the 180 target variables. The group CV model had 360 variables, 90 more than the nonrolling feature model, and the inclusion of these additional variables may have contributed to a decrease in the model’s performance [40].

### Broader Applicability and Implementation Considerations

This feasibility study employed a single platform (Apple Watch and iOS) to ensure consistency in data quality and simplify implementation. However, differences in sensors, algorithms, and available data across platforms (eg, Android and Wear OS) may lead to device-specific biases, limiting the generalizability of the findings. Future research should evaluate model performance across diverse wearable ecosystems to confirm broader applicability and ensure robustness against platform variability.

Furthermore, translating this predictive approach to clinical practice poses real-world implementation challenges. In addition to ensuring consistent user engagement and managing data gaps, practical use of consumer-grade devices must contend with technical issues such as battery life management, device malfunctions, and reliable data synchronization—all of which may affect data integrity and participant adherence. Moreover, effective integration of high-volume WD data into clinical workflows requires attention to infrastructure, data privacy, and usability concerns for both patients and clinicians. Addressing these barriers will be essential for realizing the potential of wearable-based adherence monitoring in routine care.

In addition, while psychological factors related to medication adherence were collected at baseline via a questionnaire, they were not incorporated into the current models, which focused on dynamic, time-series signals from WDs for within-individual prediction. However, these baseline psychological traits may provide complementary value in future model iterations—particularly for interindividual comparisons or hybrid models that combine static behavioral characteristics with continuous physiological data.

### Limitations

There are some limitations that need to be addressed. First, the small sample size restricted model development to within-individual analysis. Only 8 participants completed the study, and models could only be constructed for 4 participants who had a missed dose rate above 20%. This extremely small sample size limits the statistical power and generalizability of the findings. Furthermore, the demographics of this small cohort were not well-balanced, particularly concerning age distribution (50% were in their twenties, with no participants in their thirties). This skew towards younger adults, potentially influenced by the requirement of owning and using an Apple Watch and the recruitment methods employed, further limits the representativeness of our sample and the applicability of the findings to older or more diverse patient populations typically on chronic medications. Recruitment targeted patients regularly wearing smartwatches and taking medications for >3 months, as per participant eligibility. Moreover, only a subset of participants provided sufficient data for model construction. Expanding the number of participants and ensuring a more representative demographic distribution is necessary for generalization. Future studies should focus on recruiting a larger and more diverse population to confirm these preliminary findings and assess the broader applicability of the proposed approach; achieving this necessary scale and diversity may require specific strategies such as multicenter recruitment or potentially leveraging integration with larger existing wearable datasets where feasible and appropriate.

Second, the training data were limited to a 1-month period, yielding >10,000 data points per variable as time-series data. While sufficient for data collected from WDs, the variables measurable by WDs are inherently limited. Factors such as forgetting to carry medications or external disruptions (eg, work or dining out) were not captured, limiting model applicability. Although it is possible to consider methods to measure variables other than those from WD, this often involves a trade-off with patient burden and may hinder practicality [27,28]. Furthermore, the choice of zero-imputation for missing raw wearable data, while straightforward, warrants further investigation regarding potential biases, and the exploration of more sophisticated imputation techniques could be beneficial for future model development.

Third, this study did not include a control group or a direct comparison with traditional adherence monitoring methods, such as self-reporting or pill counts. The study was designed primarily as a feasibility and proof-of-concept investigation to determine if personalized predictive models could be constructed using WD data. Therefore, while our results demonstrate the potential of this approach, we cannot draw conclusions about its relative effectiveness or superiority compared to existing methods based on this study alone. Comparative studies are needed to evaluate how this WD-based approach performs with respect to established adherence monitoring techniques in terms of accuracy, cost-effectiveness, and patient and provider burden.

Another limitation is that this study did not examine the potential influence of specific medication characteristics, such as drug type, indication, regimen complexity, or experienced side effects, on adherence patterns or model performance. To reduce participant burden and facilitate recruitment for this 30-day observational study involving WDs and daily app reporting, we intentionally limited the scope of collected medication-related data. However, these factors are well-known to significantly impact medication adherence, and their interaction with lifestyle patterns measured by WDs warrants investigation.

Finally, external validation was not performed. Variability among individuals and differences in WD hardware limited interindividual analysis. Additionally, in models aimed at monitoring long-term adherence, even for the same individual, the patient’s lifestyle may gradually change, potentially rendering the initial training data ineffective [26]. Future studies should address these issues through external validation and real-world testing.

### Conclusions

This study developed and compared 2 predictive models, the group CV and nonrolling feature models, to identify the occurrence of missed medication events using WD data. Overall, both models demonstrated high precision, confirming that there were few false detections of missed medication events. However, challenges in predicting afternoon events revealed the limitations of WD-measured variables. Our study also highlighted the relevance of physiological and lifestyle changes in the hours preceding missed doses, emphasizing the relationship between daily routines and medication adherence. Future studies should focus on expanding participant numbers, conducting external validation, and refining models to enhance recall for critical medications. These findings may pave way for developing a robust system to monitor and improve patients’ medication adherence in clinical settings.

## Supplementary material

10.2196/72113 Multimedia Appendix 1 Data preprocessing and analysis.

10.2196/72113 Multimedia Appendix 2 Code for prediction model.

10.2196/72113 Multimedia Appendix 3 Individual event occurrence rates.

10.2196/72113 Multimedia Appendix 4 Feature importance.

10.2196/72113 Checklist 1 Consolidated Reporting Guidelines for Prognostic and Diagnostic Machine Learning Modeling Studies.

## Abbreviations

CV: cross-validation
LightGBM: Light Gradient Boosting Machine
WD: wearable device
