# Supplementary material for: Construction of Personalized Predictive Models for Missed Medication Doses Using Wearable Device Data: Prospective Observational Study
Source: JMIR Form Res. 2025 Jun 24;9:e72113. doi: 10.2196/72113 (PMC12212888; doi:10.2196/72113)
Supplement: Multimedia Appendix 1 [file formative-v9-e72113-s001.docx]

**Appendix1 Data Preprocessing and Analysis**

**1-1. Visualization of Time-Series Data Types**

Description: The recorded time series data is divided into three-minute intervals, and contains data for [3 minutes x 480] x [number of recorded days].

Therefore, each chart has rows for the number of days of recorded data (30 days). Each row displays one day's worth of data, and from the left of each column, it represents data for the time periods [00:00-00:02], [00:03-00:05], ... [23:57-23:59]. If data exists, a green bar is drawn, and if data is missing, nothing is drawn. The darkness of the green indicates the size of the data.

*Exercise Time*


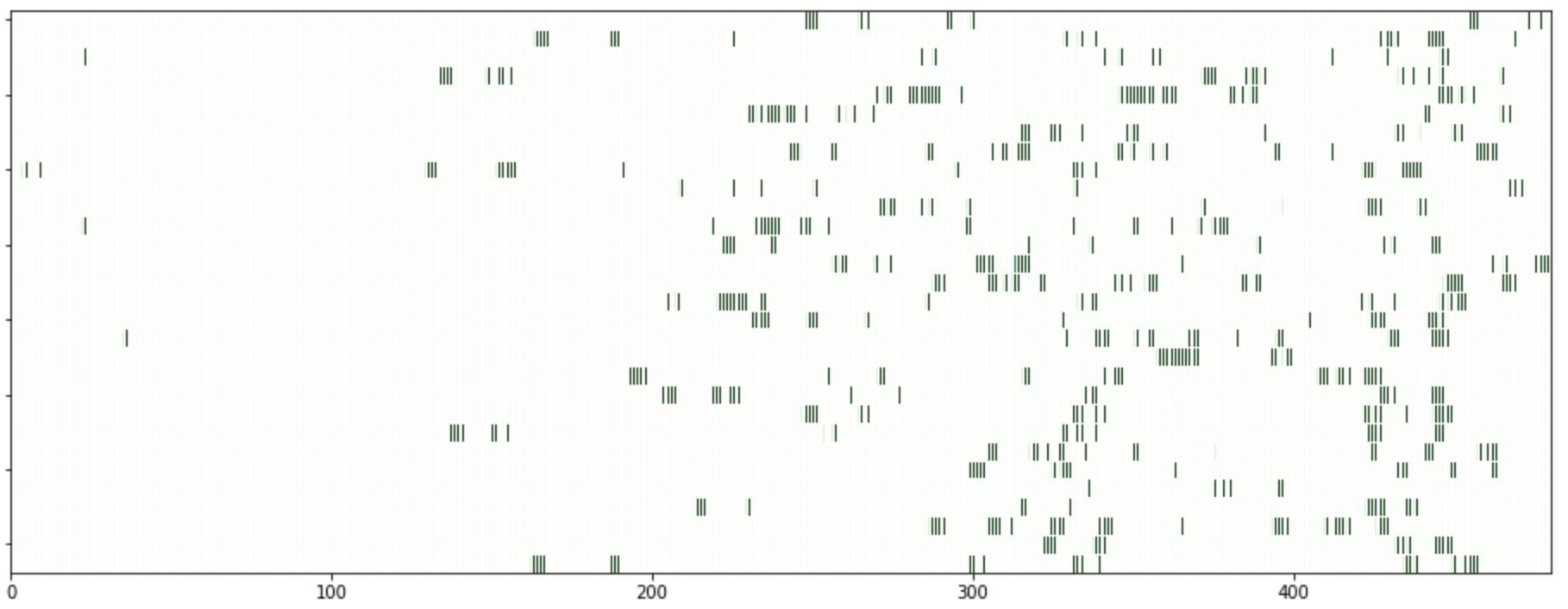


*Resting Heart Rate*


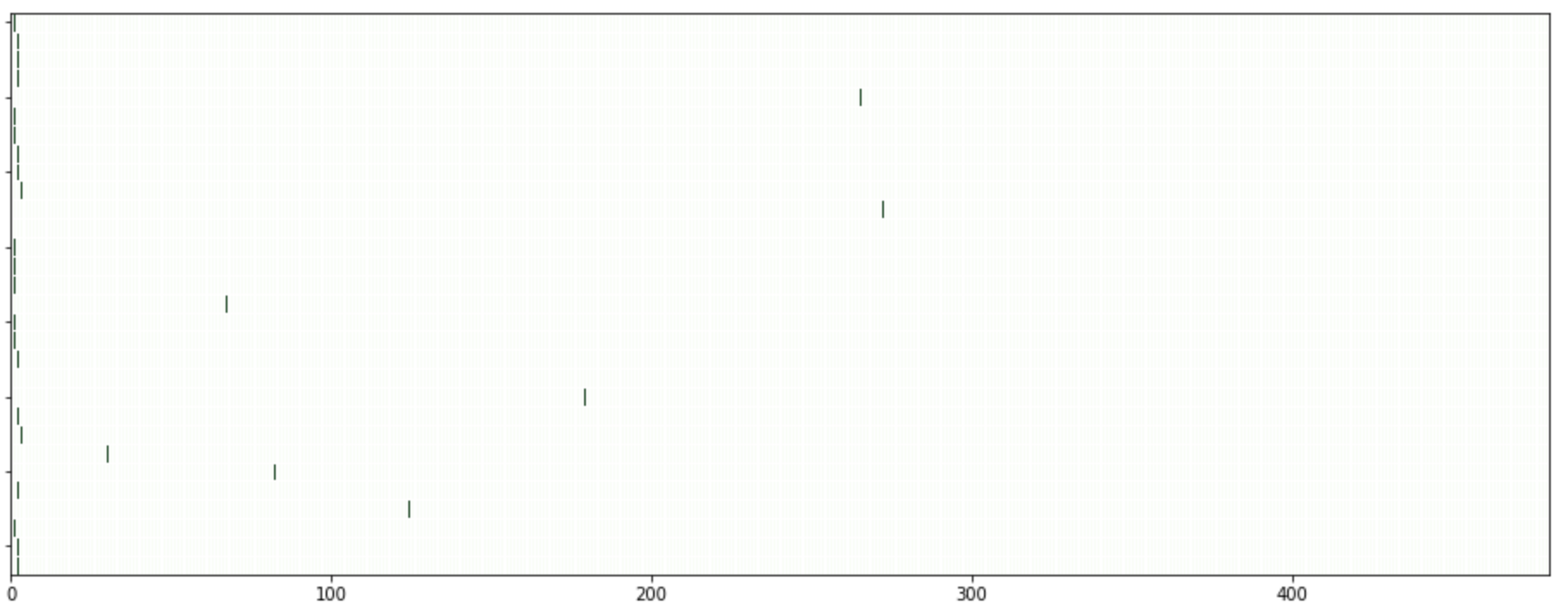


*Active Energy Burned*


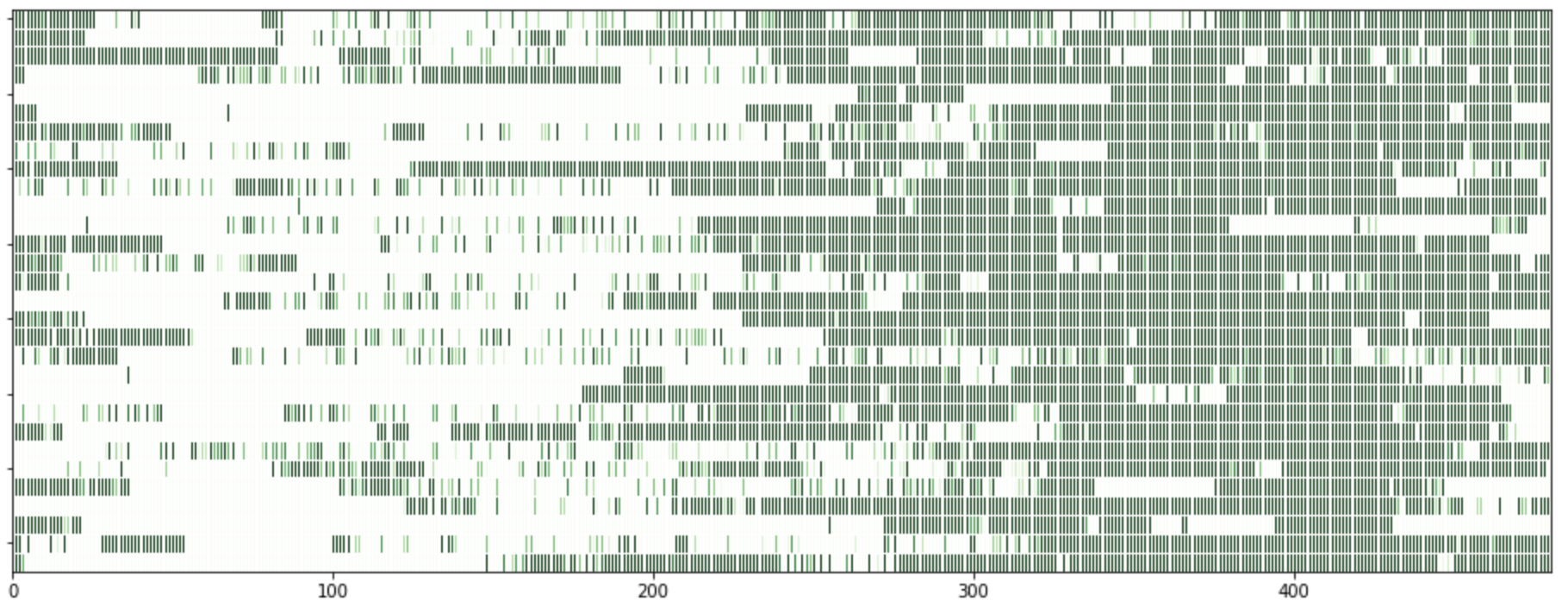


*Step Count*


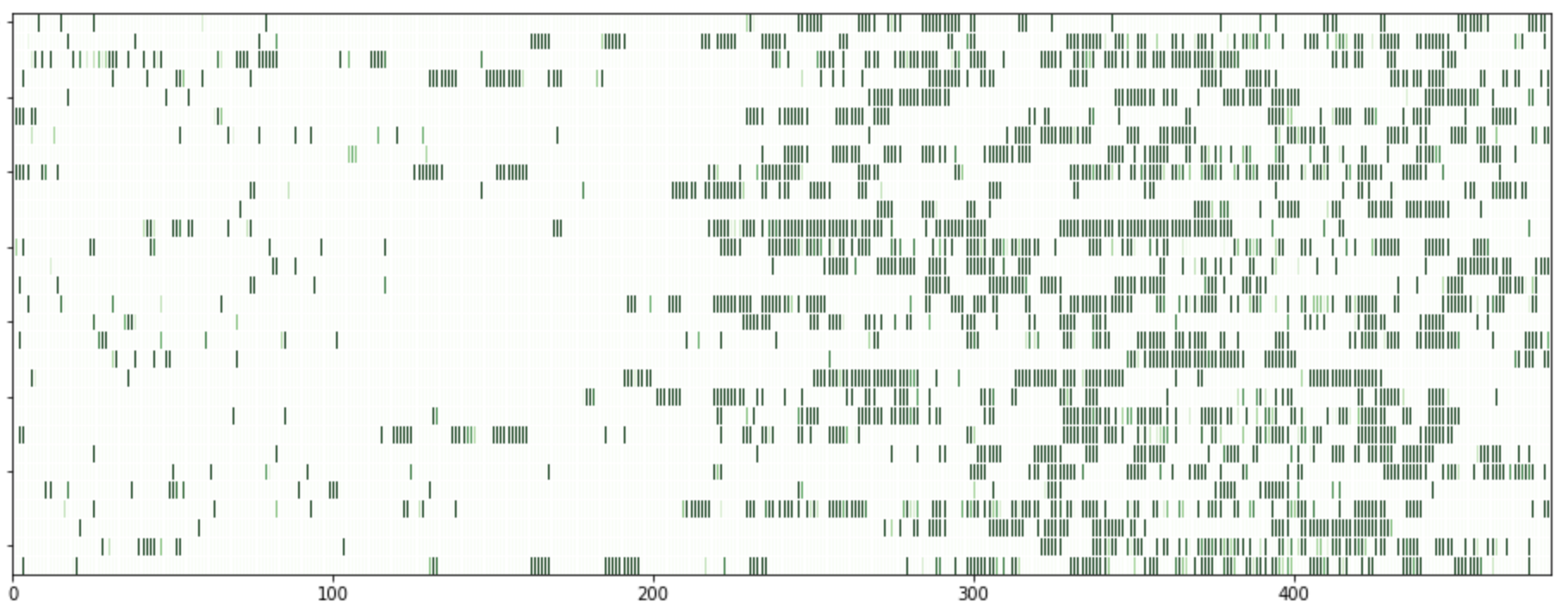


*Oxygen Saturation*


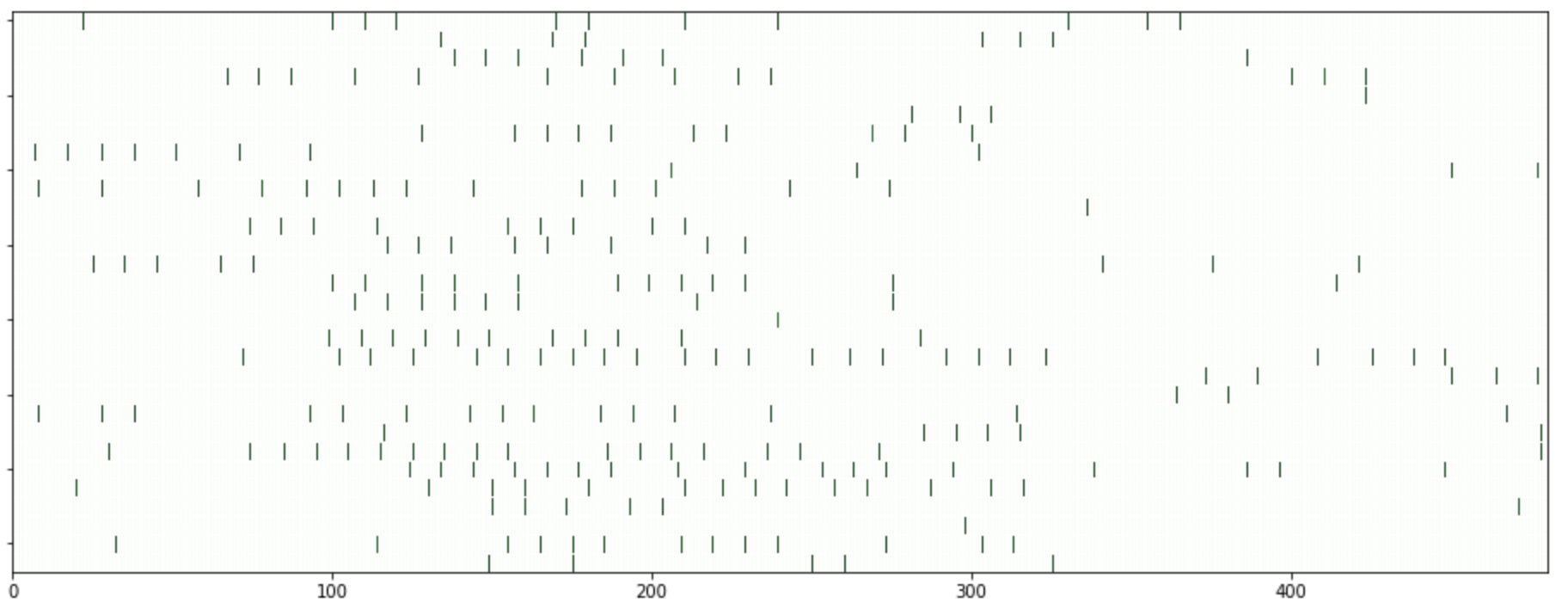


*Walking Heart Rate Average*

*
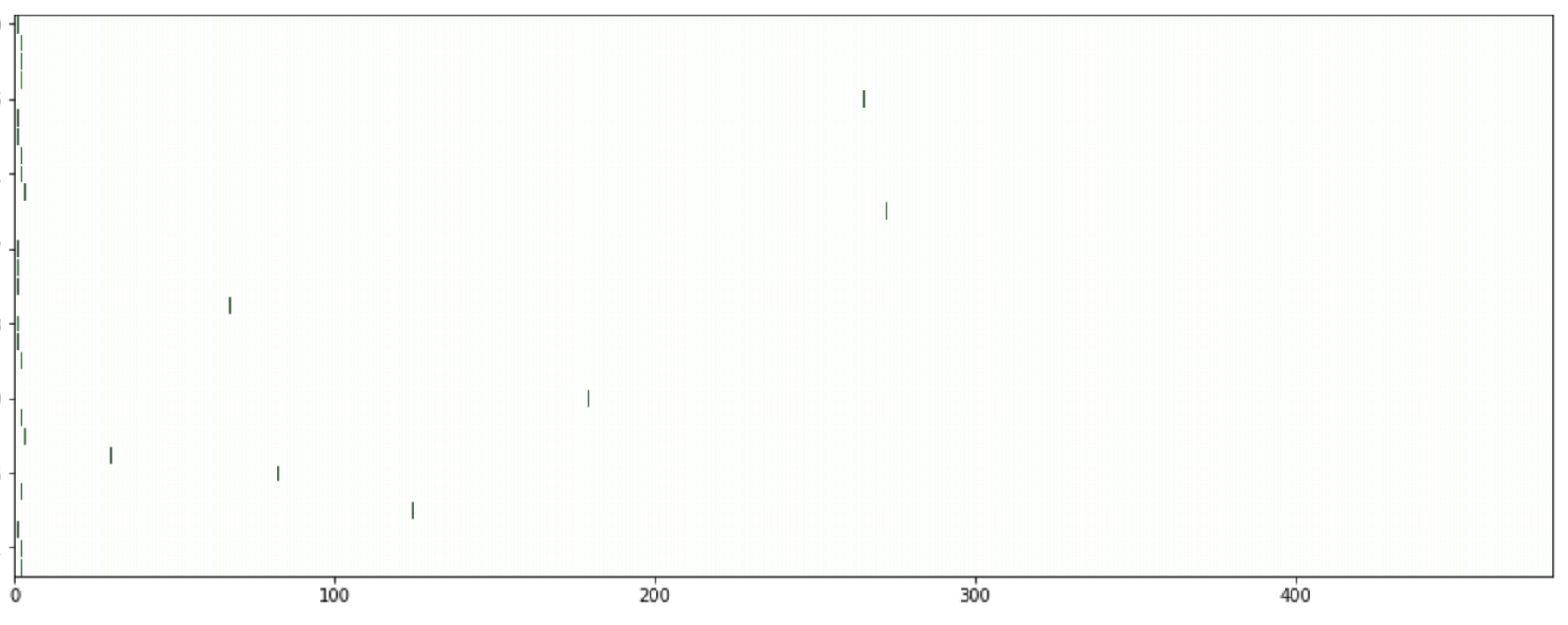
*

*Walking Double Support Percentage*

*
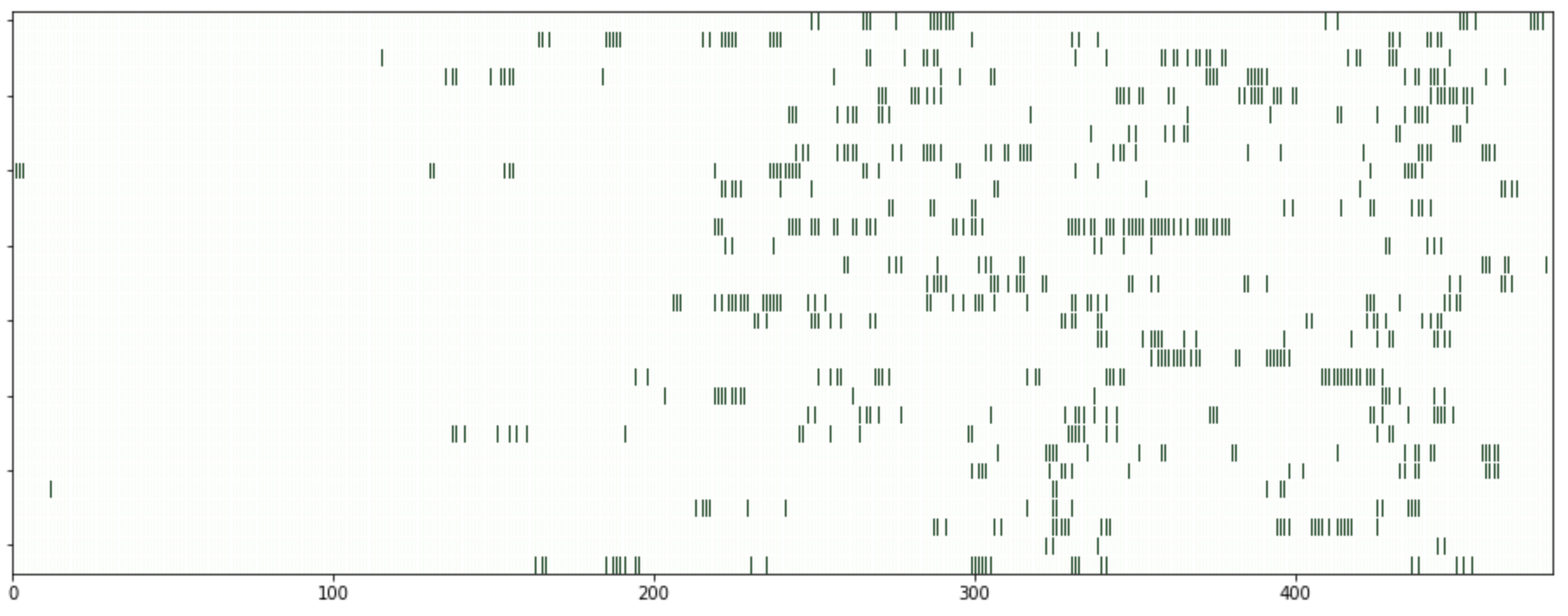
*

*Stair Descent Speed*

*
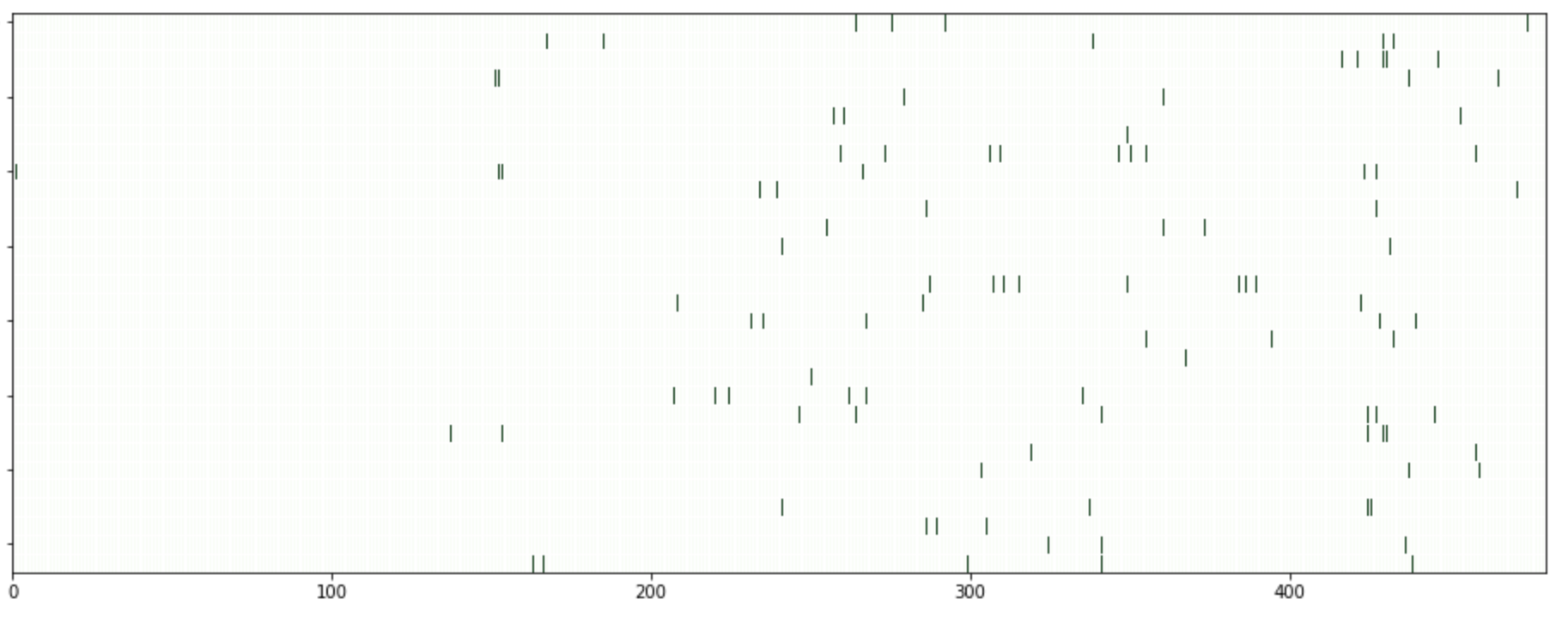
*

*Stair Ascent Speed*

*
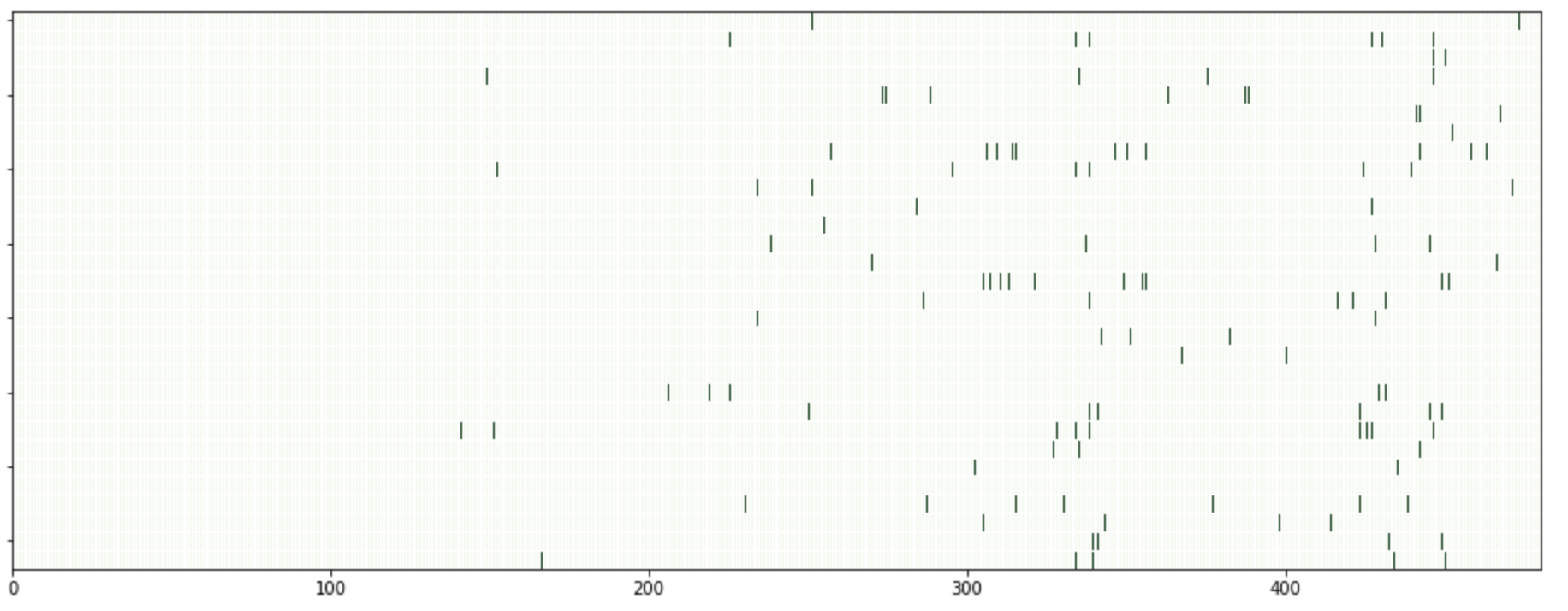
*

*VO2 Max*


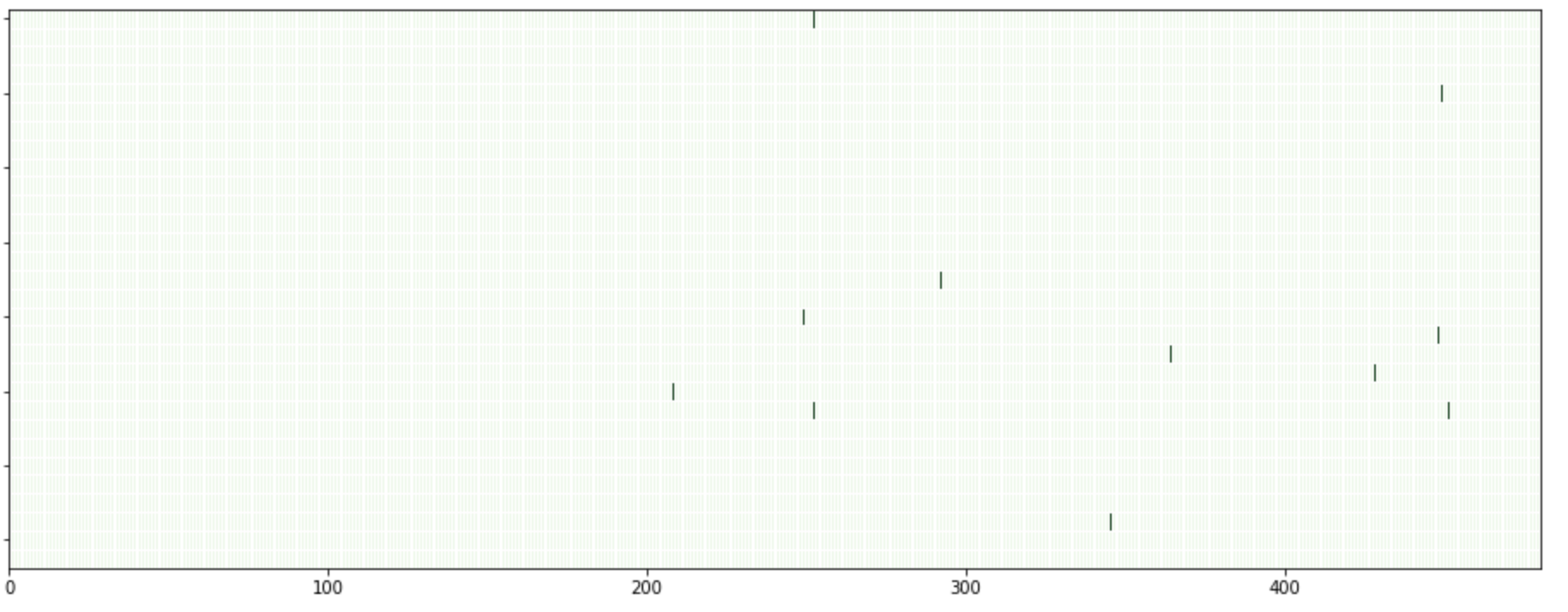


*Basal Energy Burned*

*
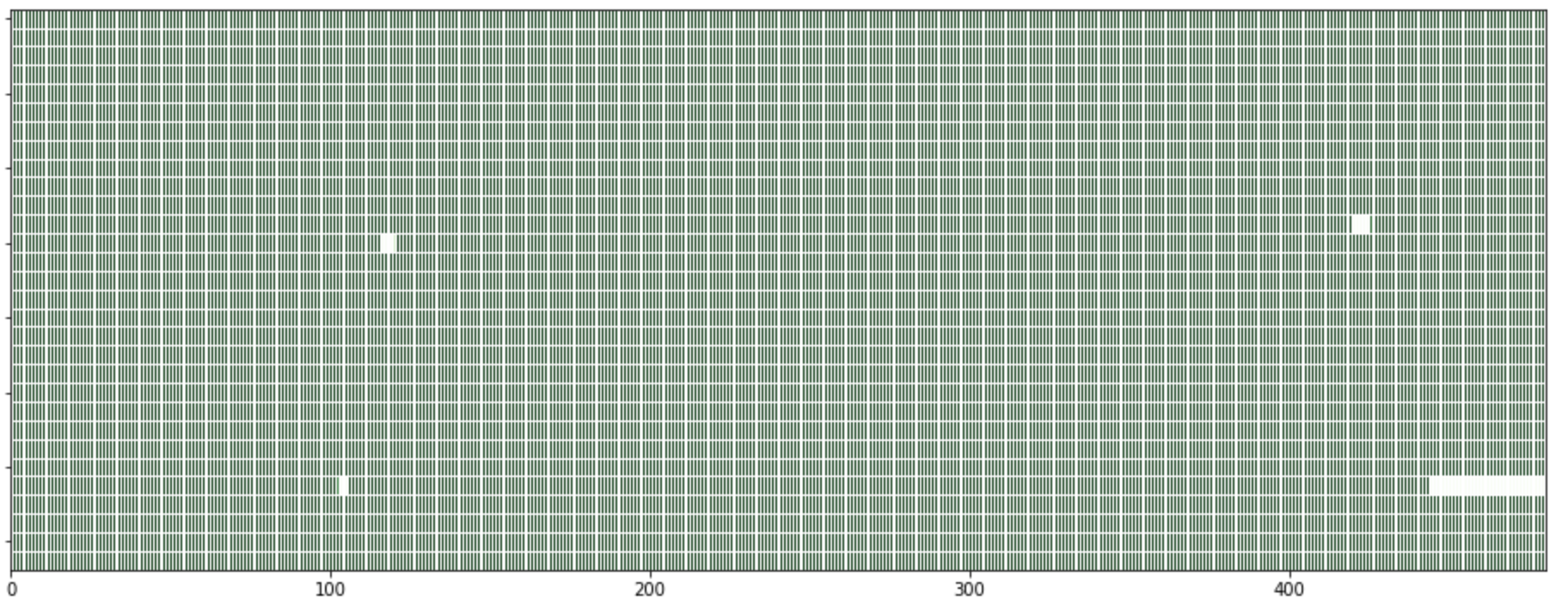
*

*Six Minute Walk Test Distance*

*
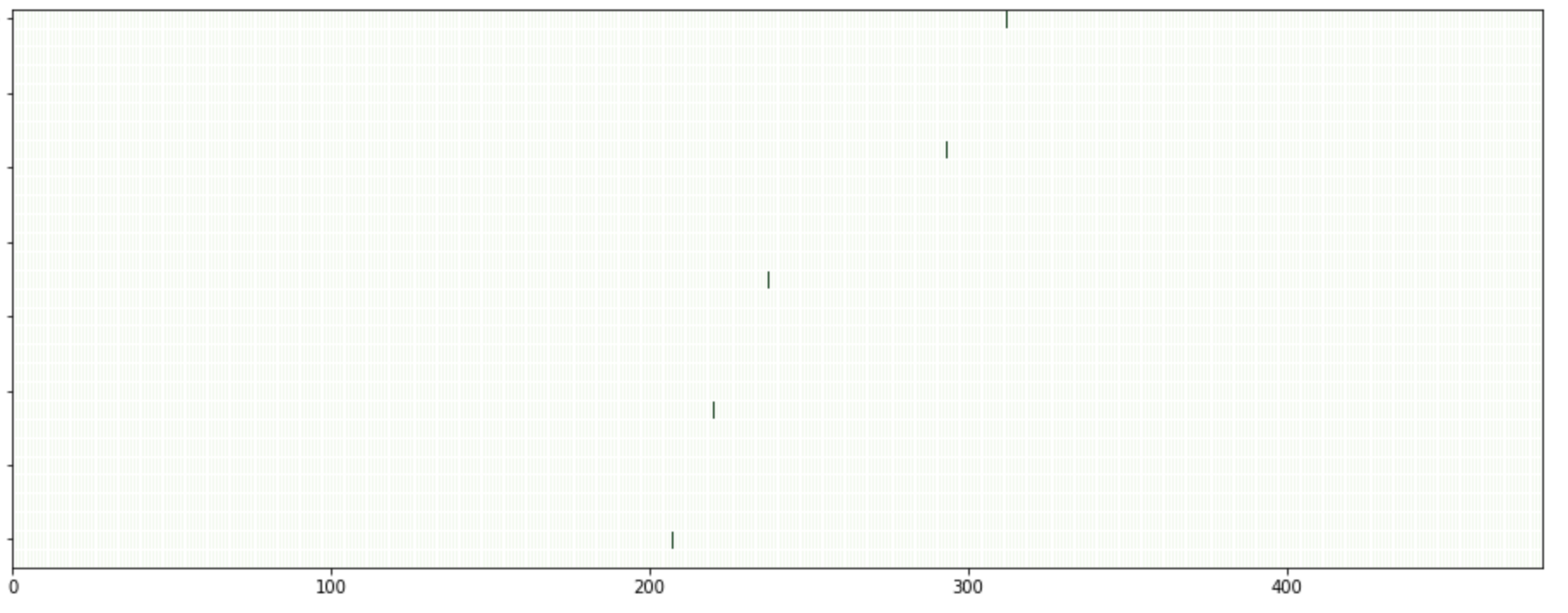
*

*Walking Speed*


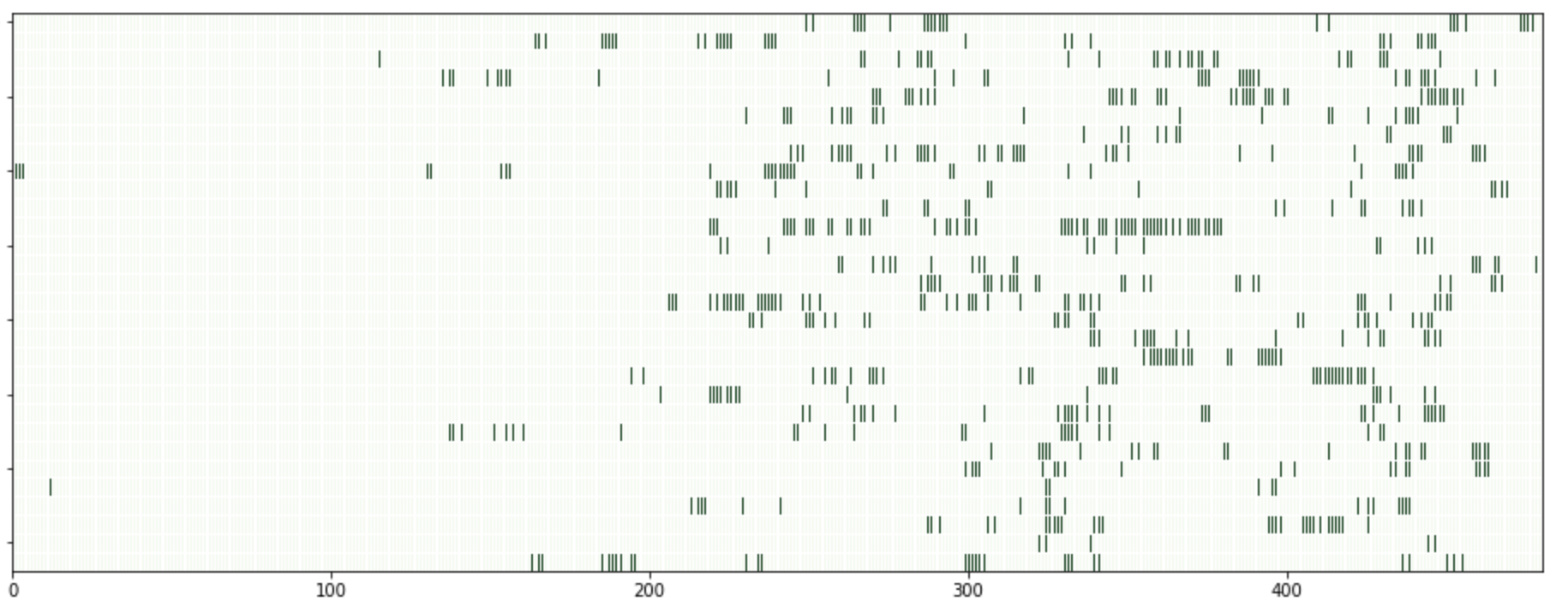


*Distance Walking Running*

*
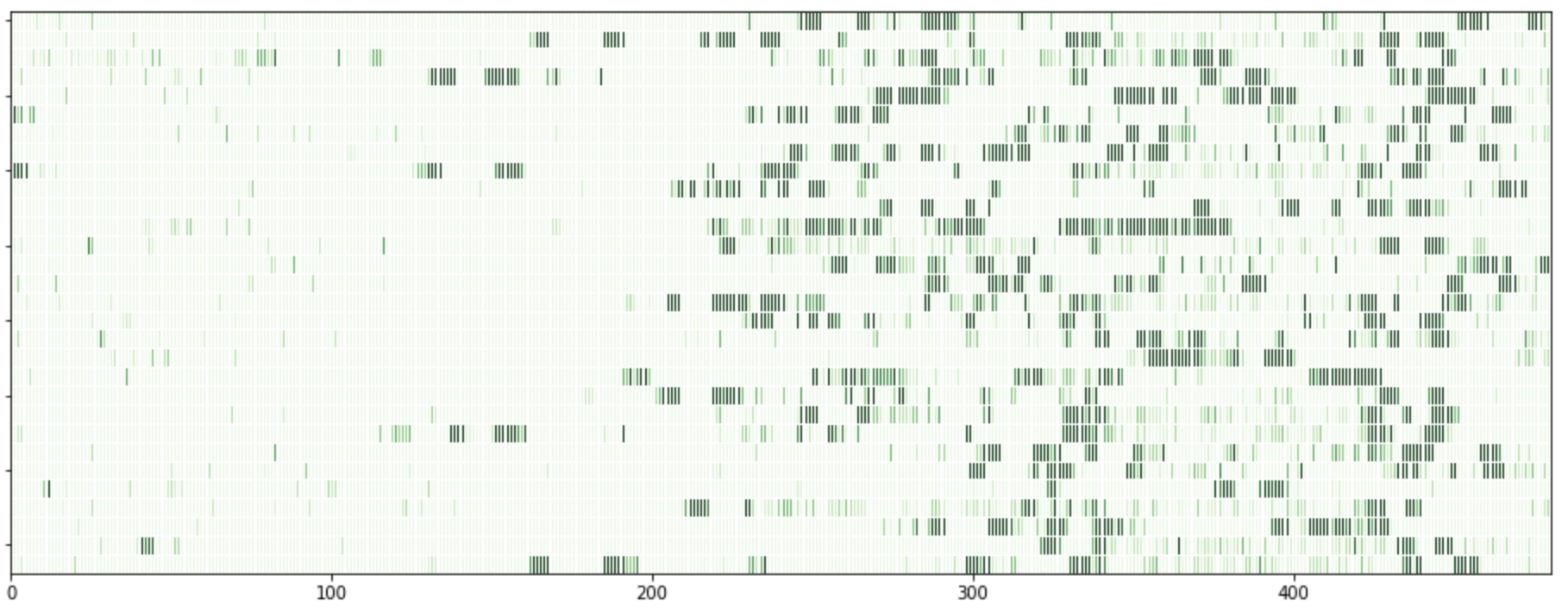
*

*Flights Climbed*

*
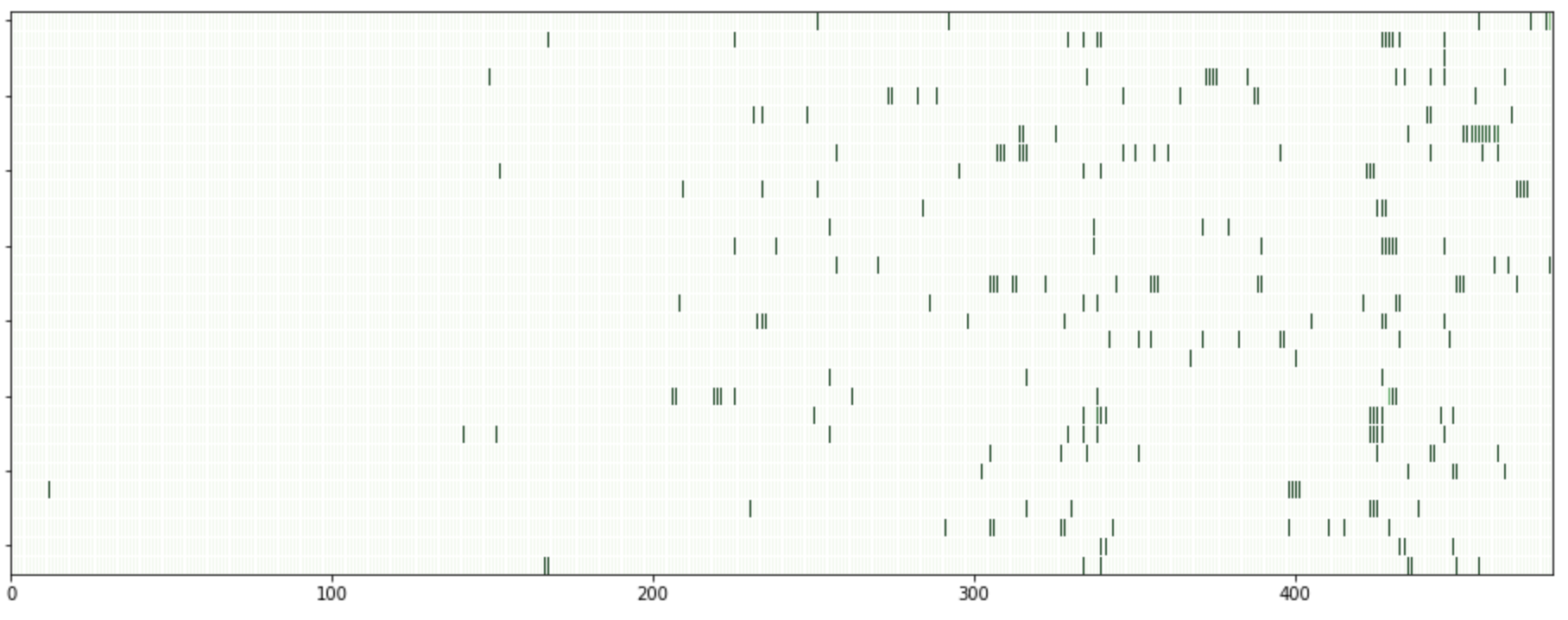
*

*Walking Step Length*


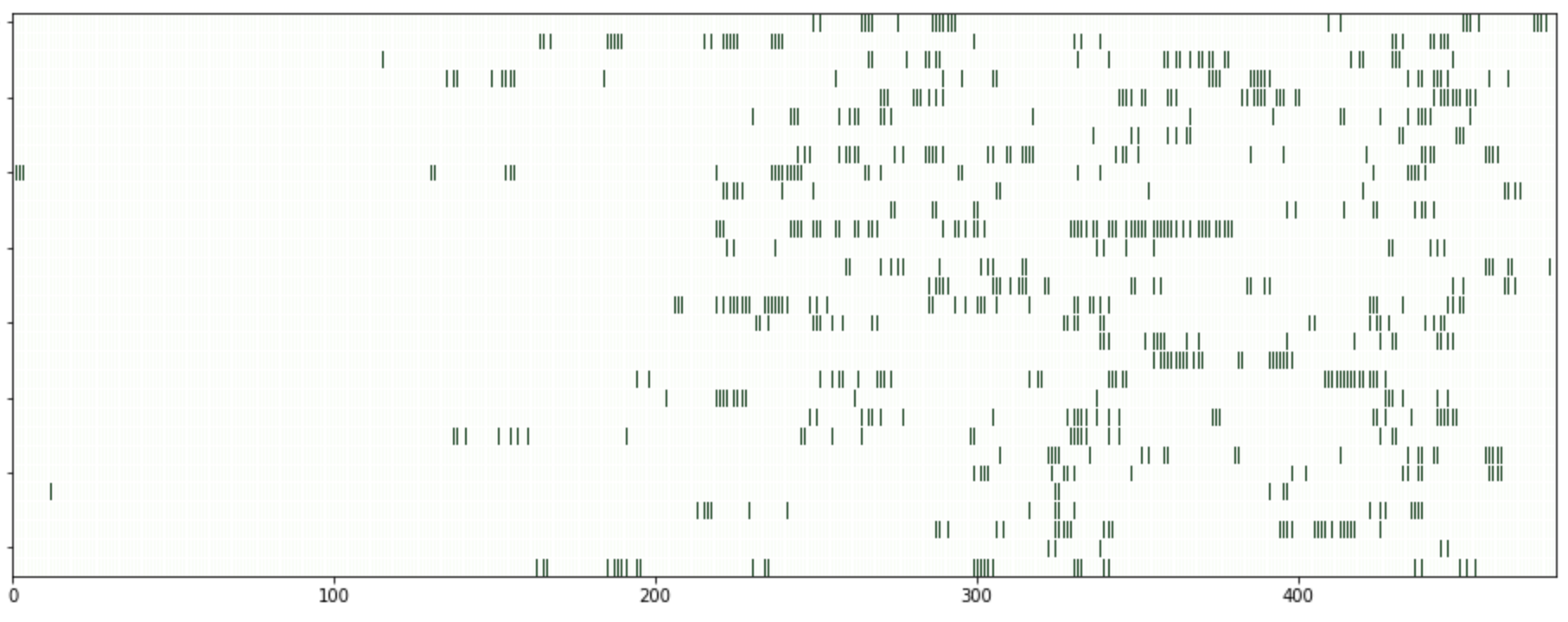


*Stand Time*

*
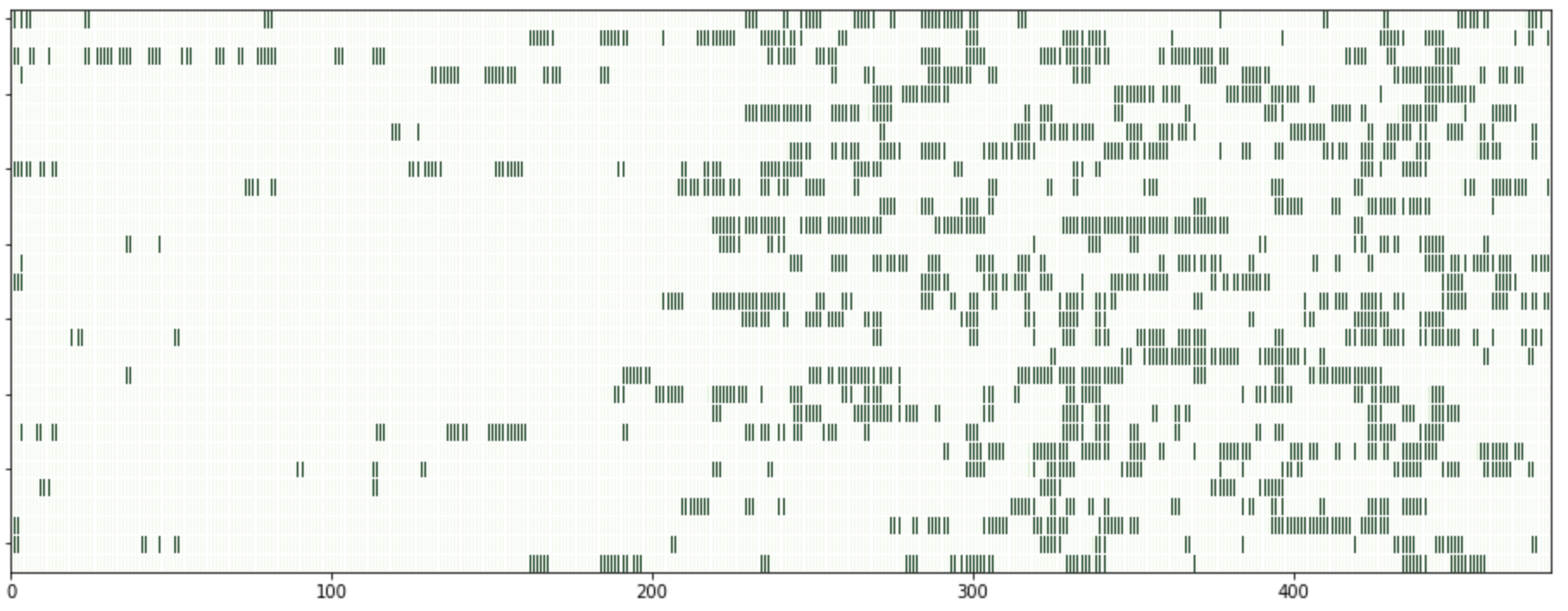
*

*Environmental Audio Exposure*

*
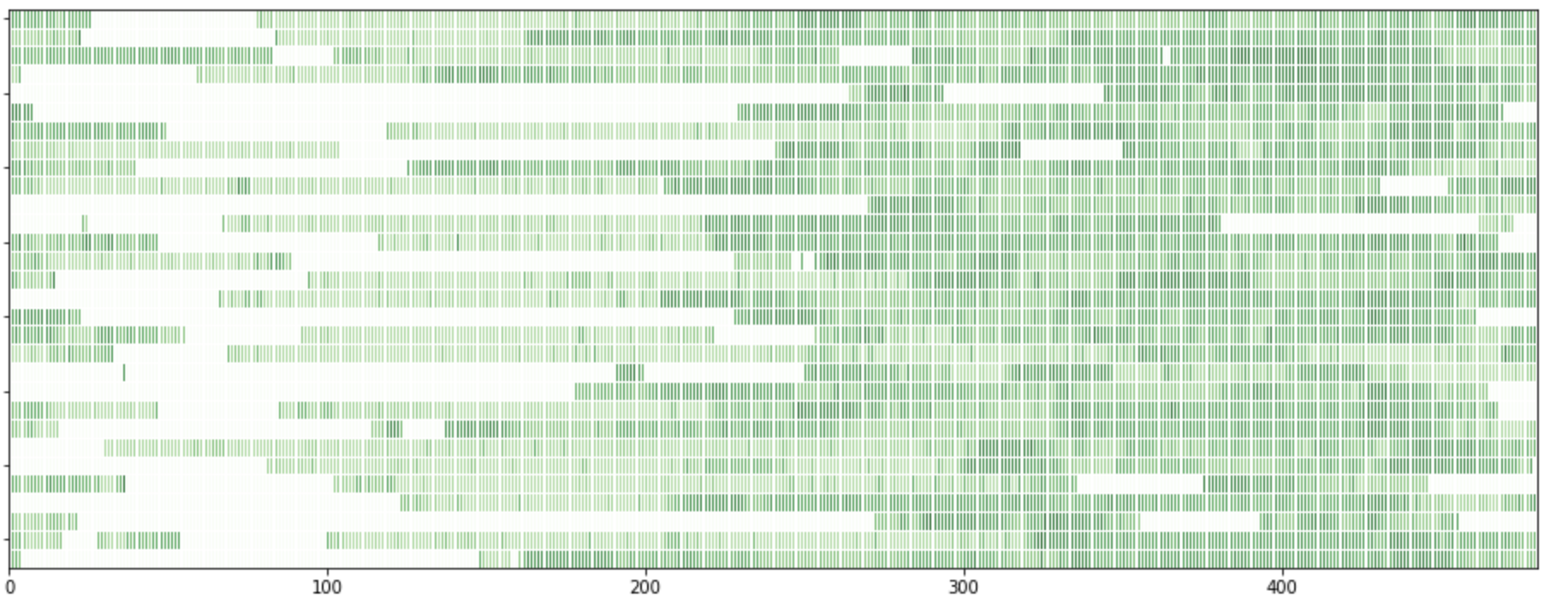
*

*Walking Asymmetry Percentage*

*
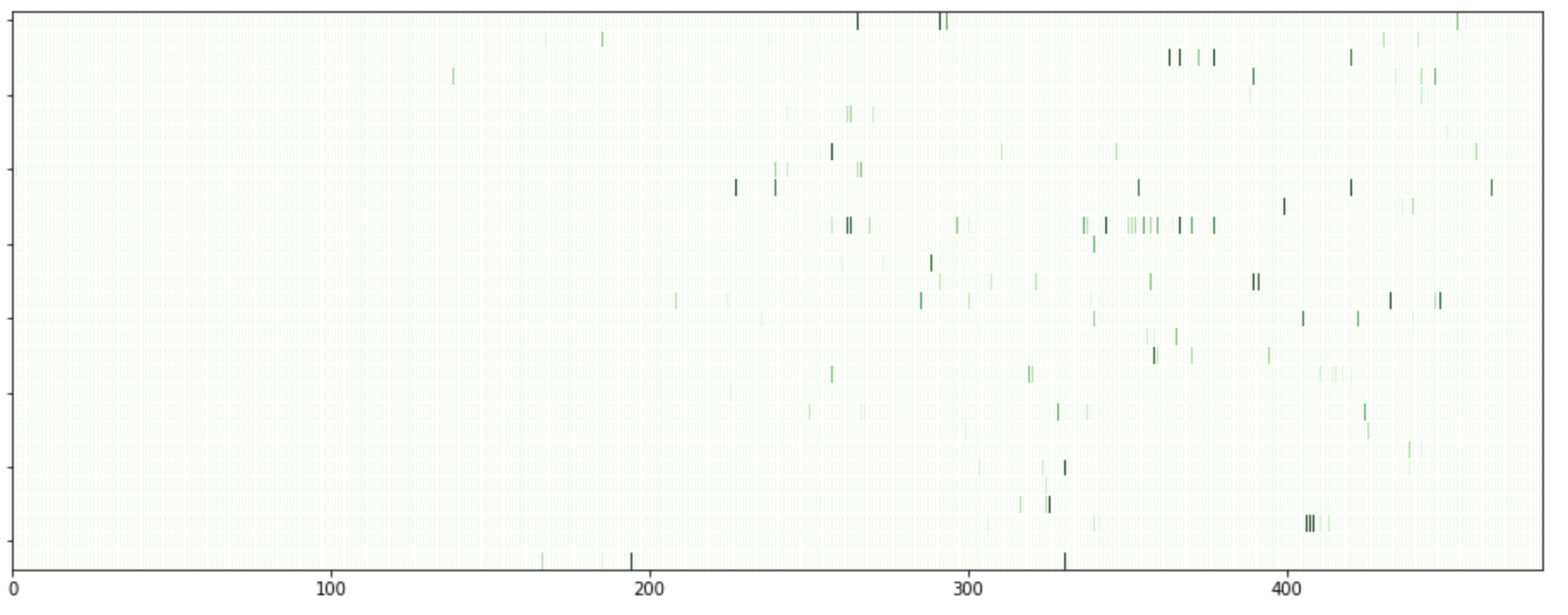
*

*Heart Rate Variability SDNN*

*
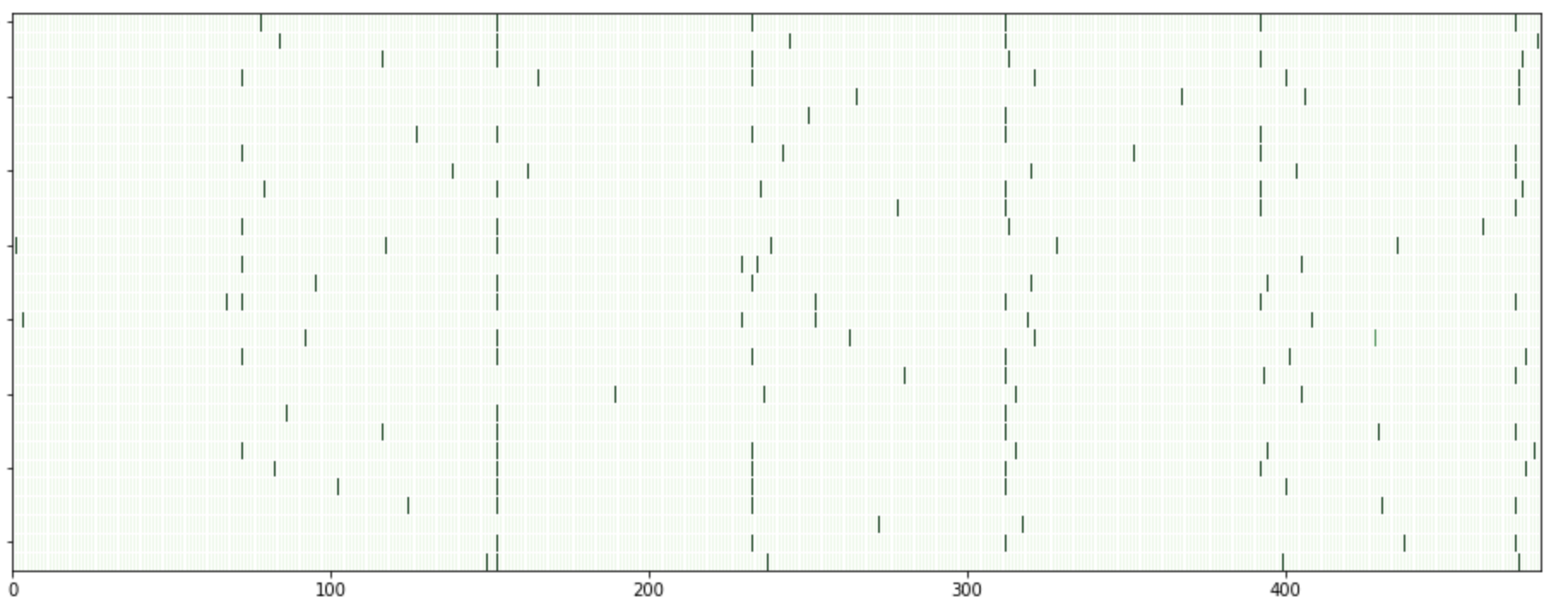
*

*Heart Rate*

*
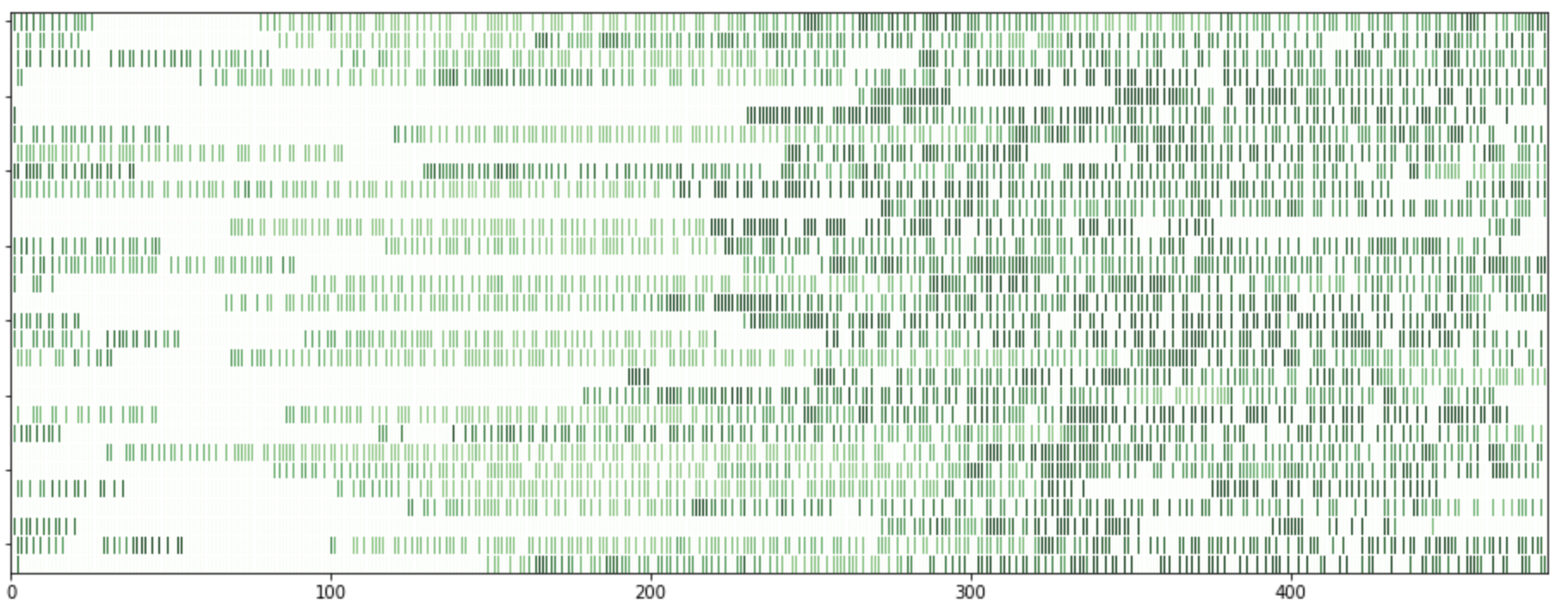
*

**1-2. Additive Decomposition of Time-Series Components**

Description: additive decomposition was performed based on the additive model. Each box represents, from top to bottom, the original data, trend component, seasonal component, and residual.

Note: Component analysis was not performed for items that were deemed to have insufficient data in 1-1.

*Exercise Time*


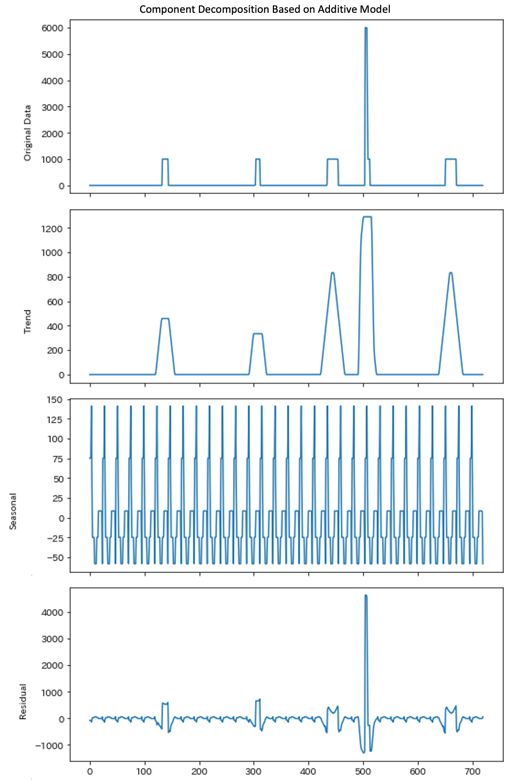


*Resting Heart Rate*


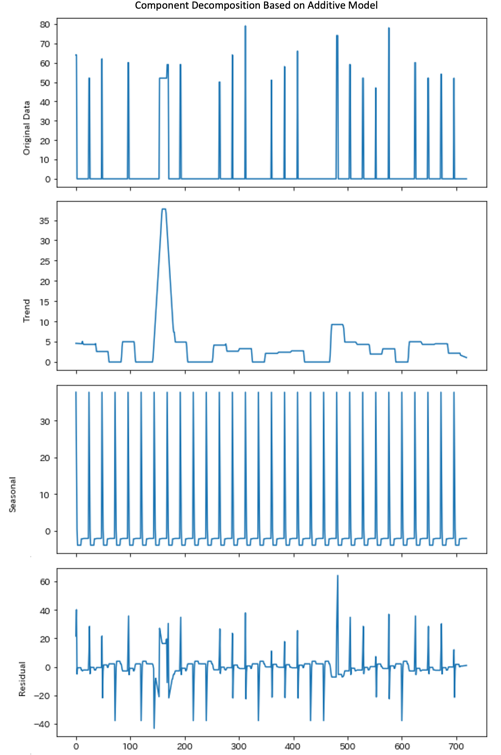


*Active Energy Burned*


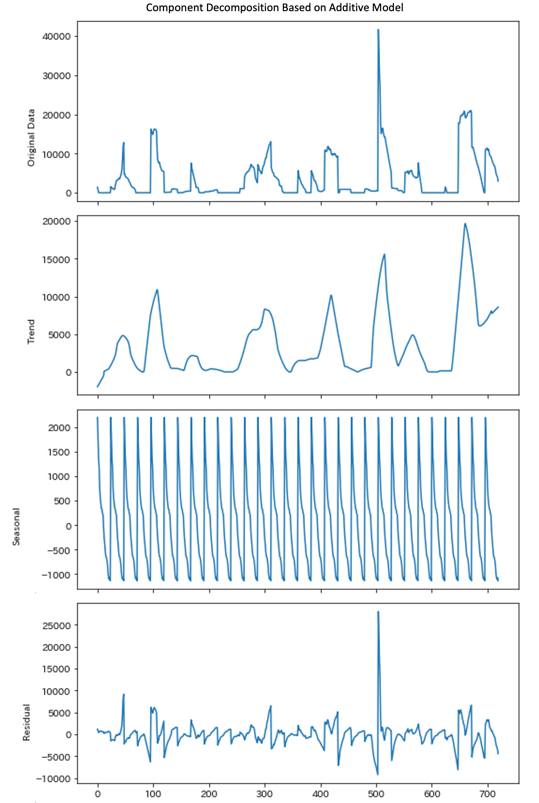


*Step Count*


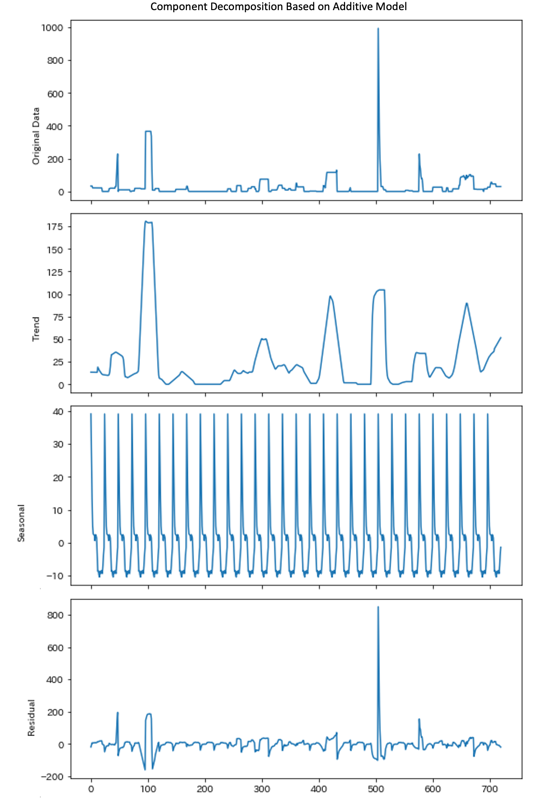


*Oxygen Saturation*


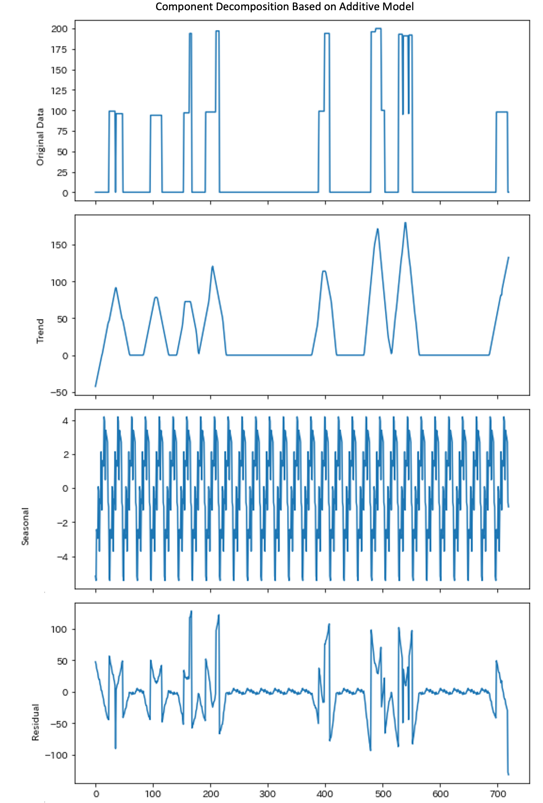


*Walking Heart Rate Average*

*
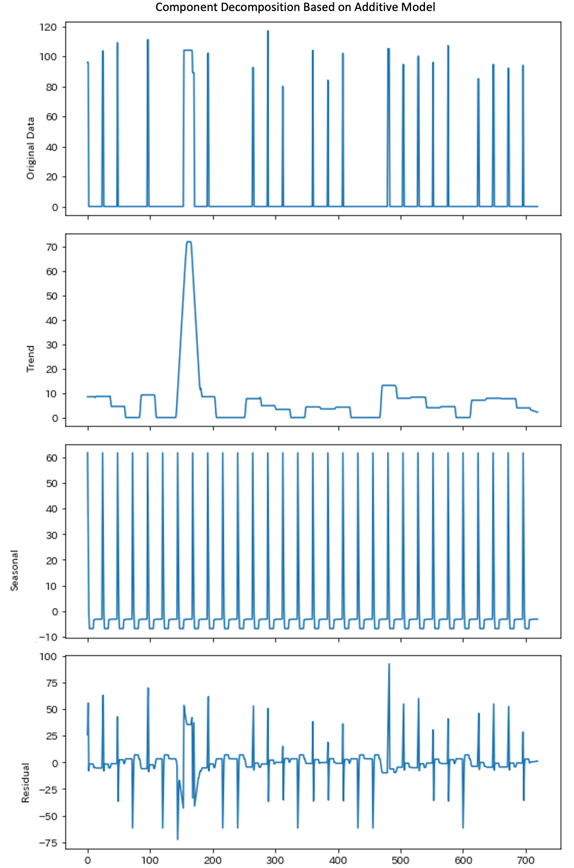
*

*Walking Double Support Percentage*

*
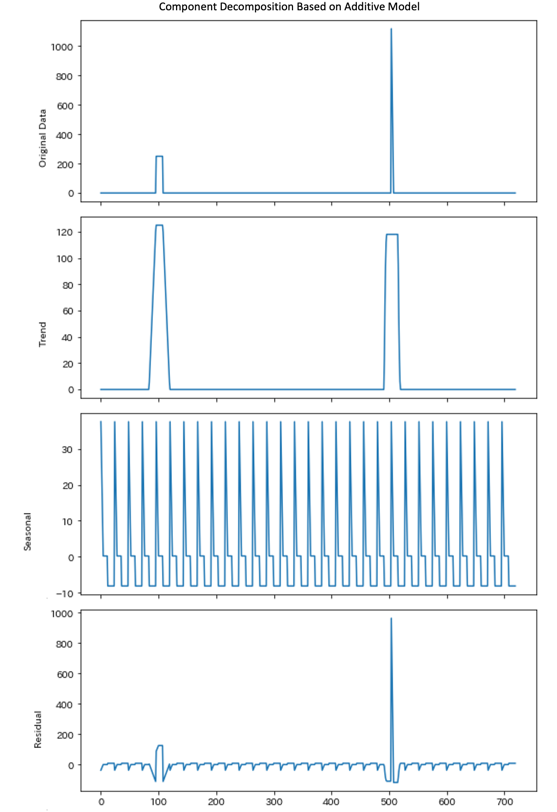
*

*Basal Energy Burned*

*
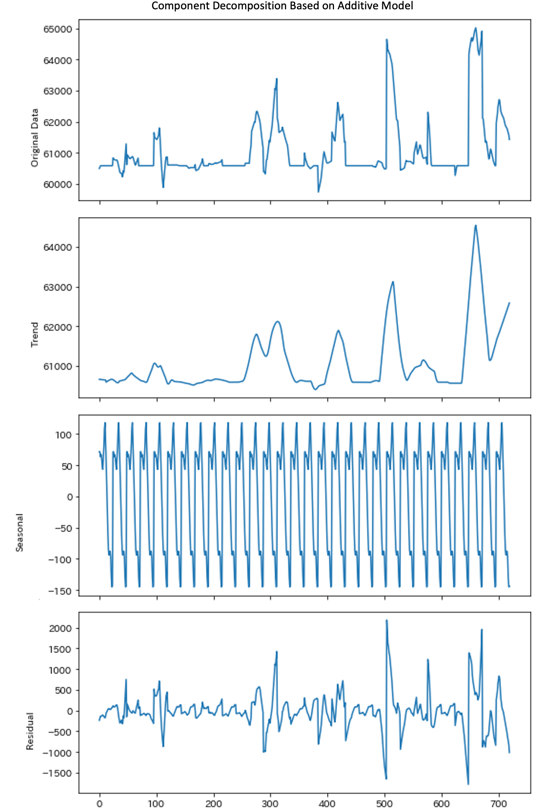
*

*Walking Speed*


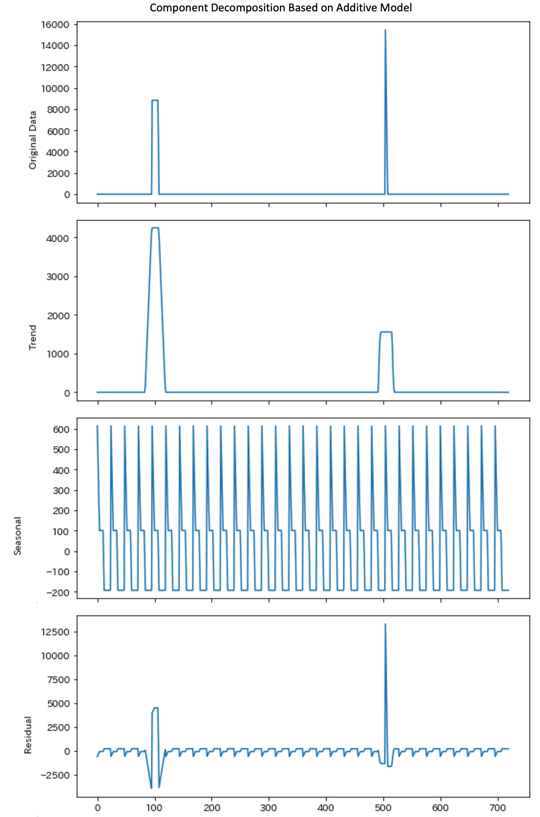


*Distance Walking Running*

*
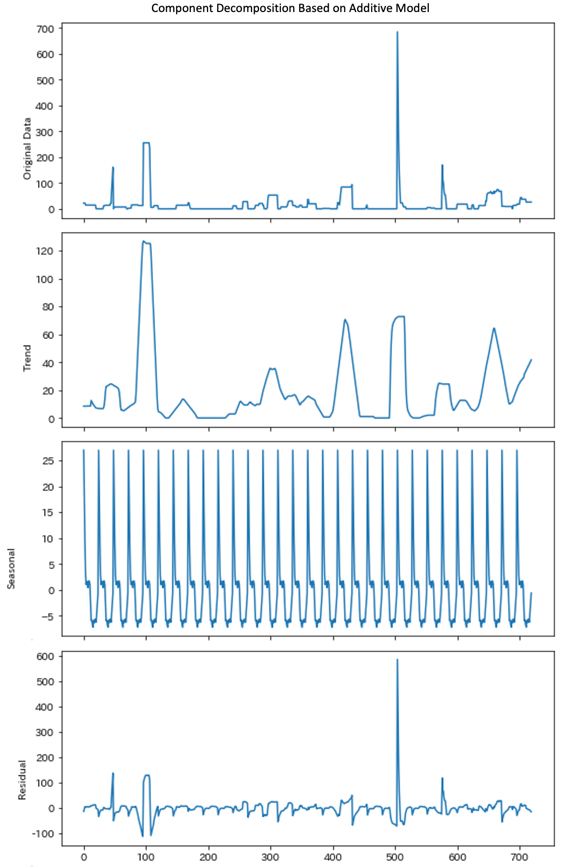
*

*Flights Climbed*

*
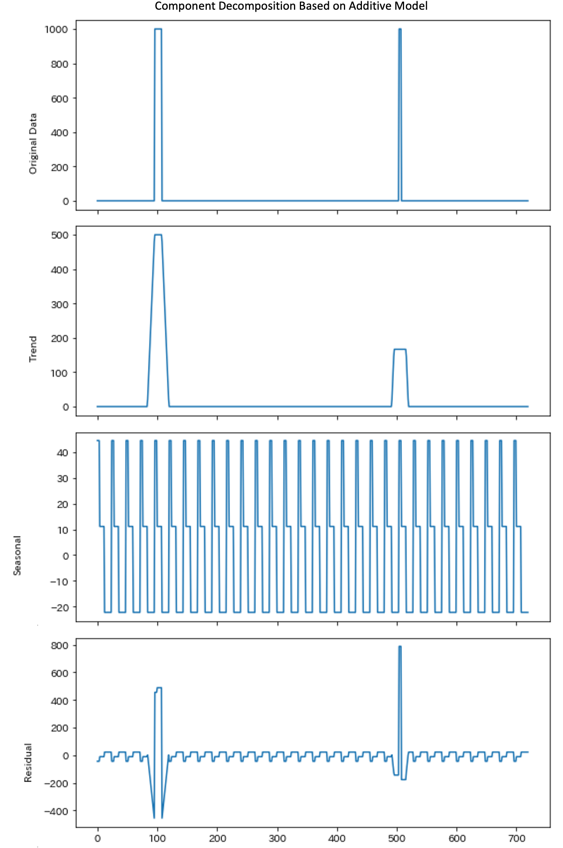
*

*Walking Step Length*


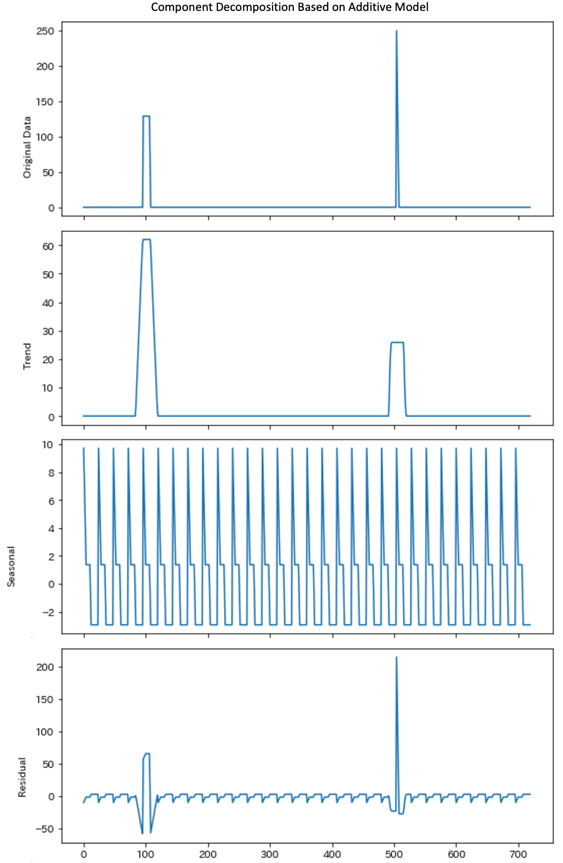


*Stand Time*

*
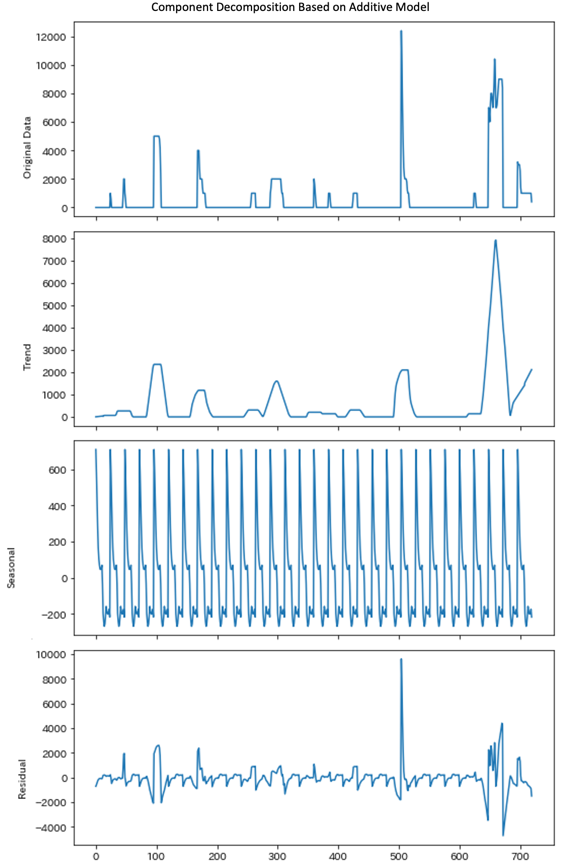
*

*Environmental Audio Exposure*

*
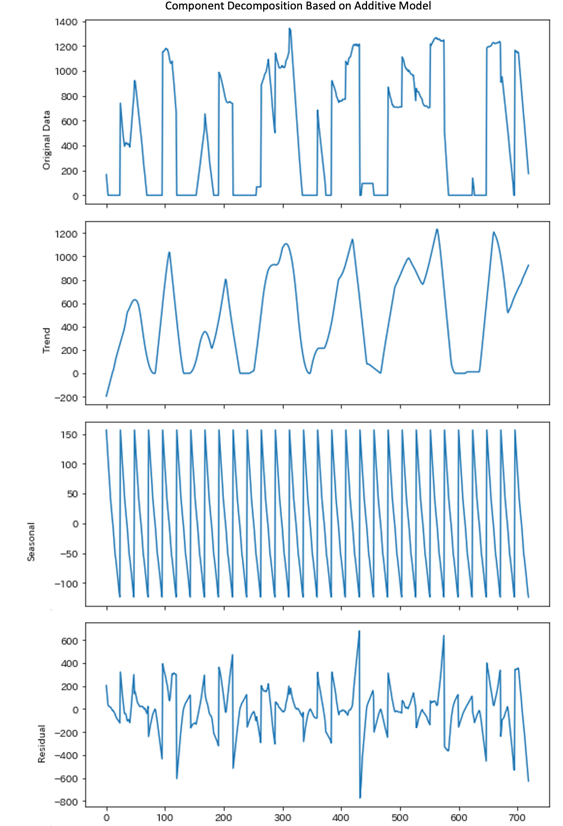
*

*Heart Rate*

*
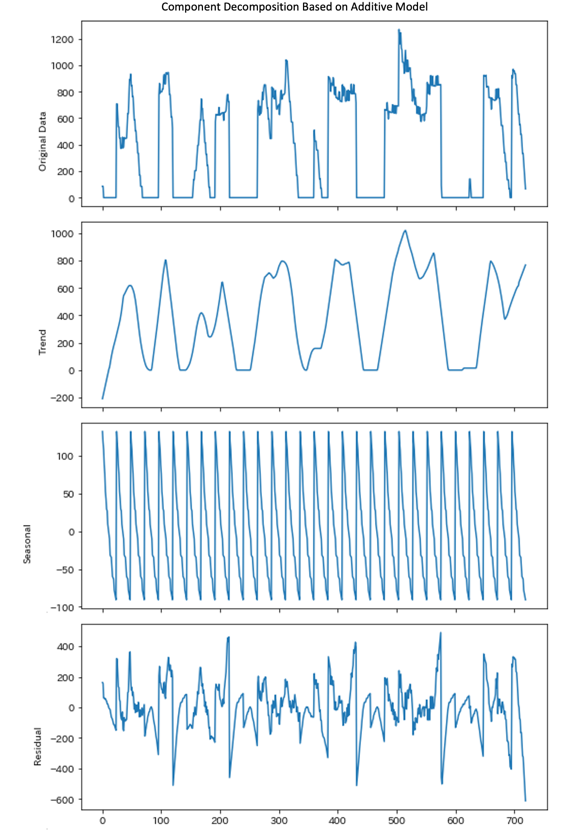
*

**1-3. Spectral Analysis of Time-Series Data using Fast Fourier Transform**

Note: Spectral analysis was not performed for items that were deemed to have insufficient data in 1-1.

*Exercise Time*


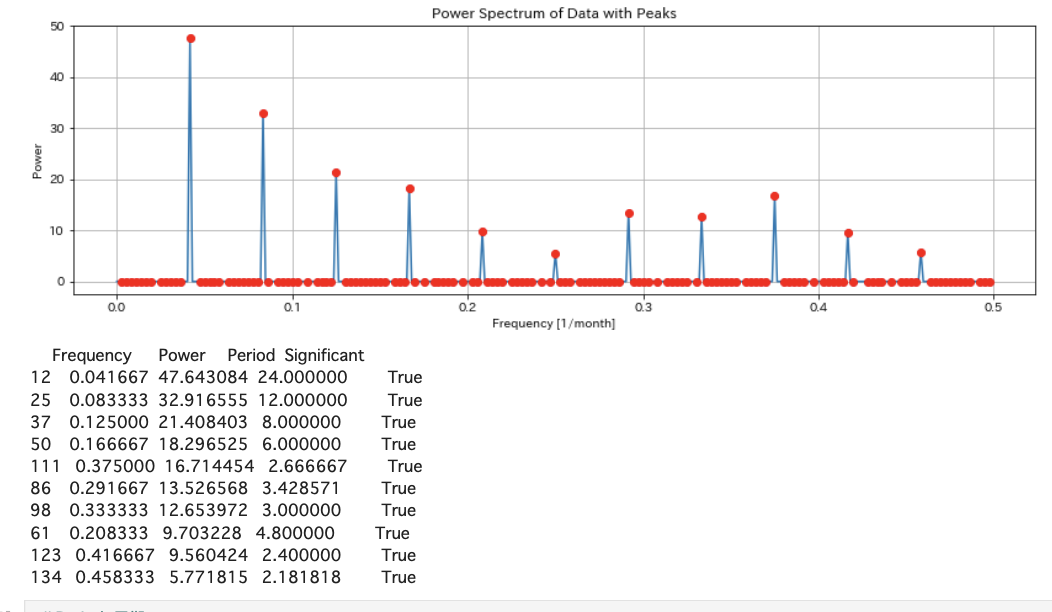


*Resting Heart Rate*


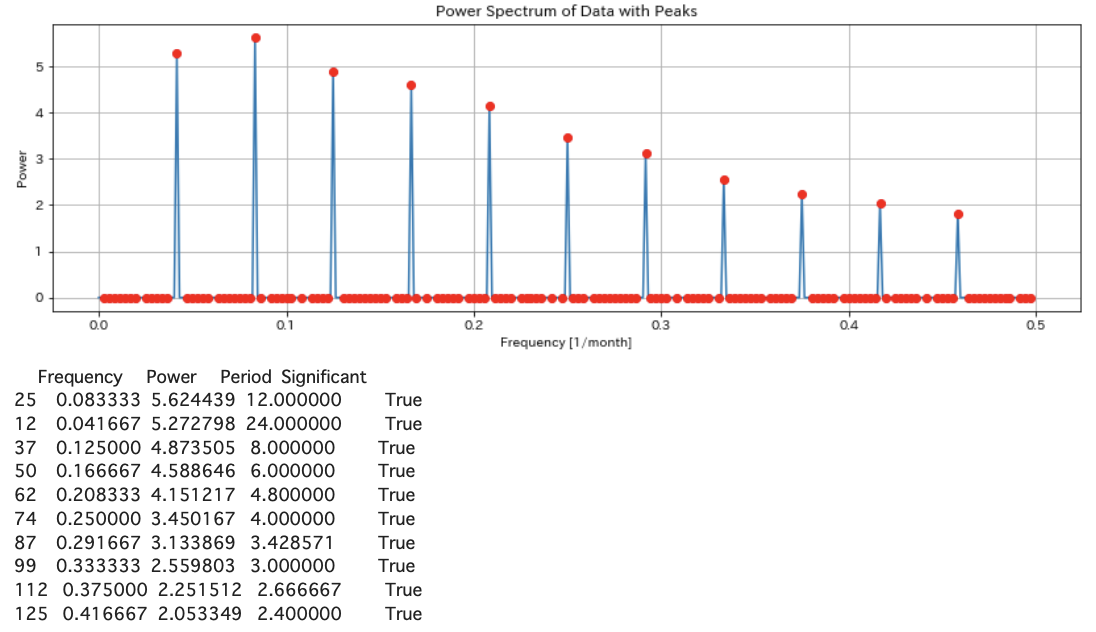


*Active Energy Burned*


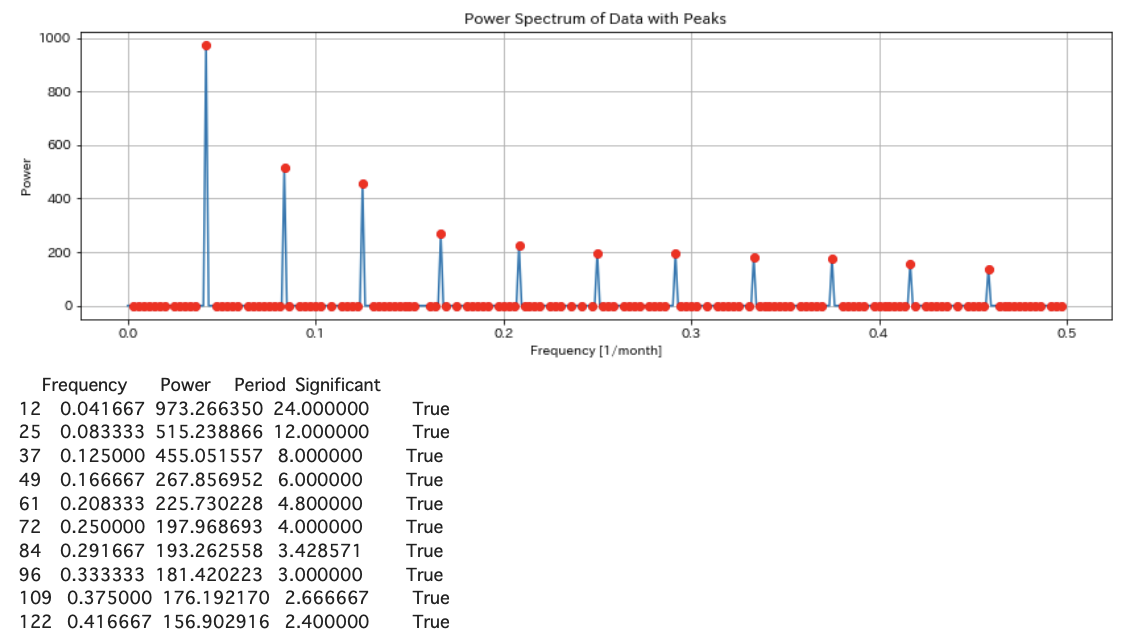


*Step Count*


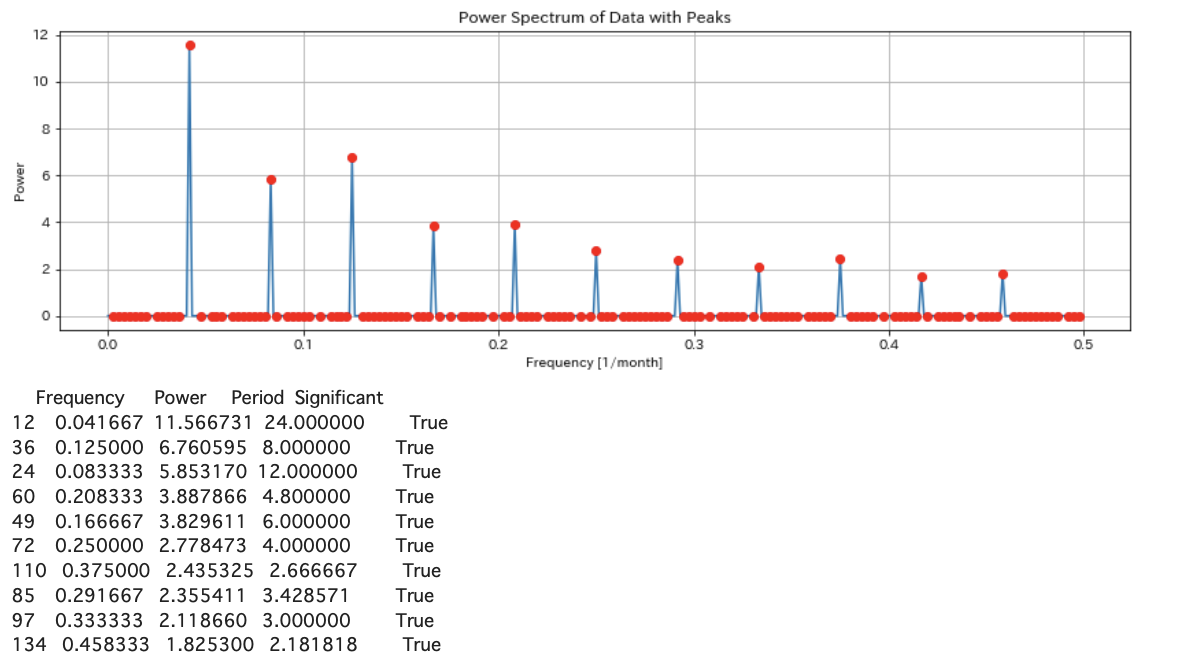


*Oxygen Saturation*


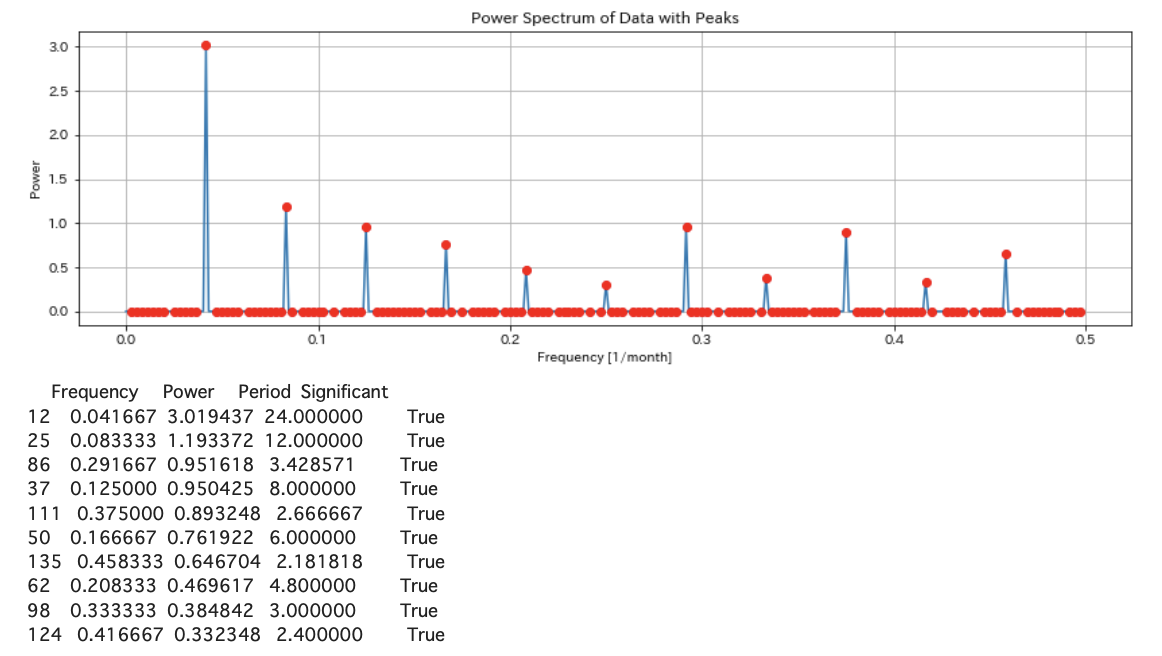


*Walking Heart Rate Average*

*
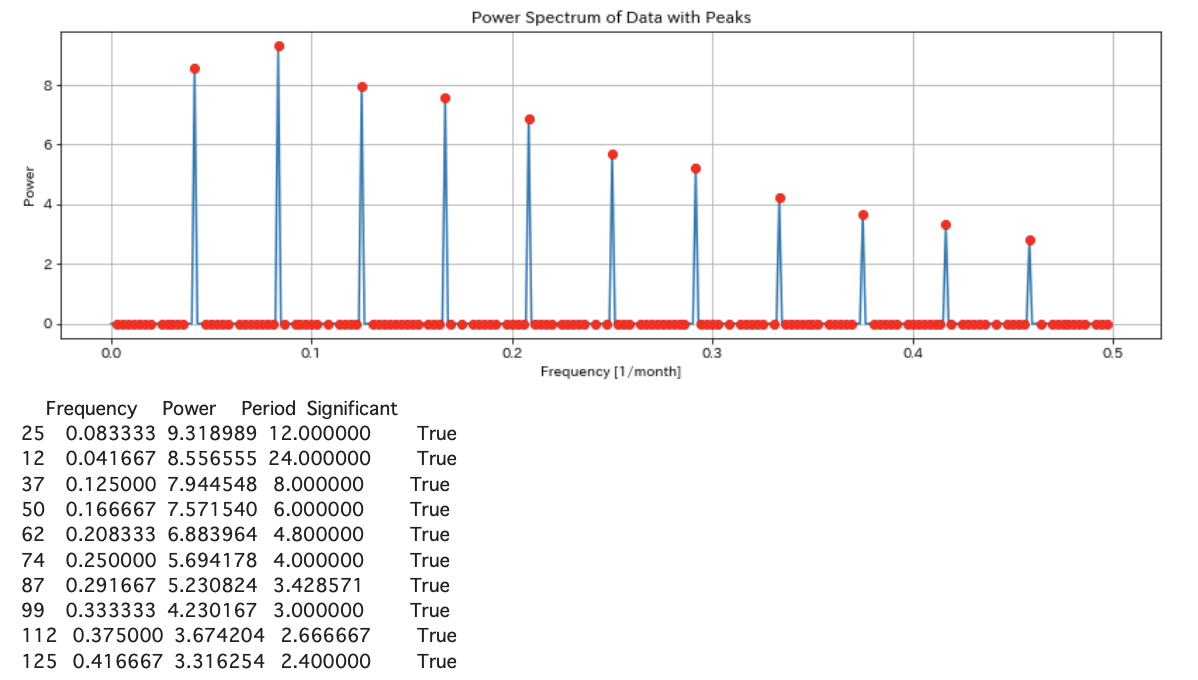
*

*Walking Double Support Percentage*

*
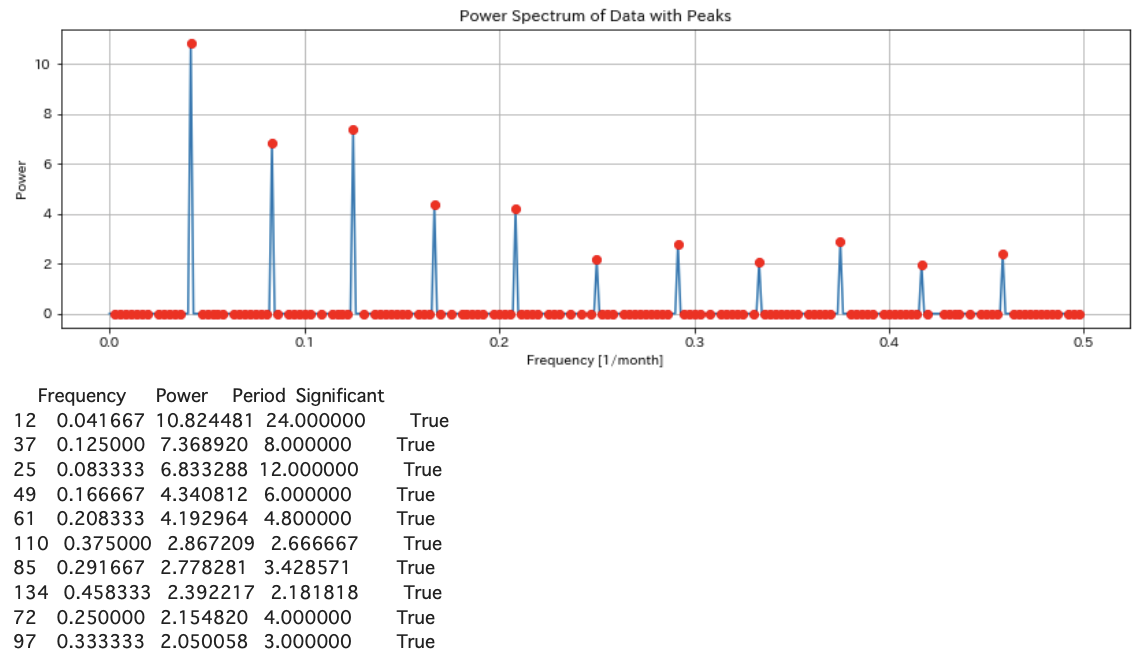
*

*Basal Energy Burned*

*
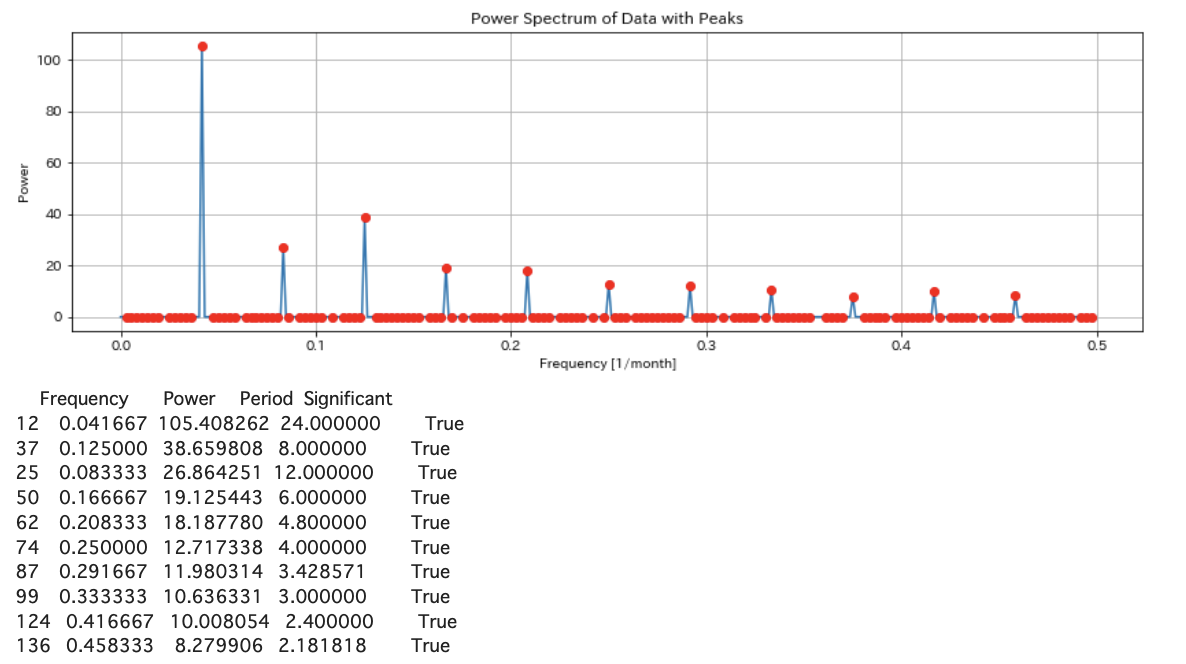
*

*Walking Speed*

*
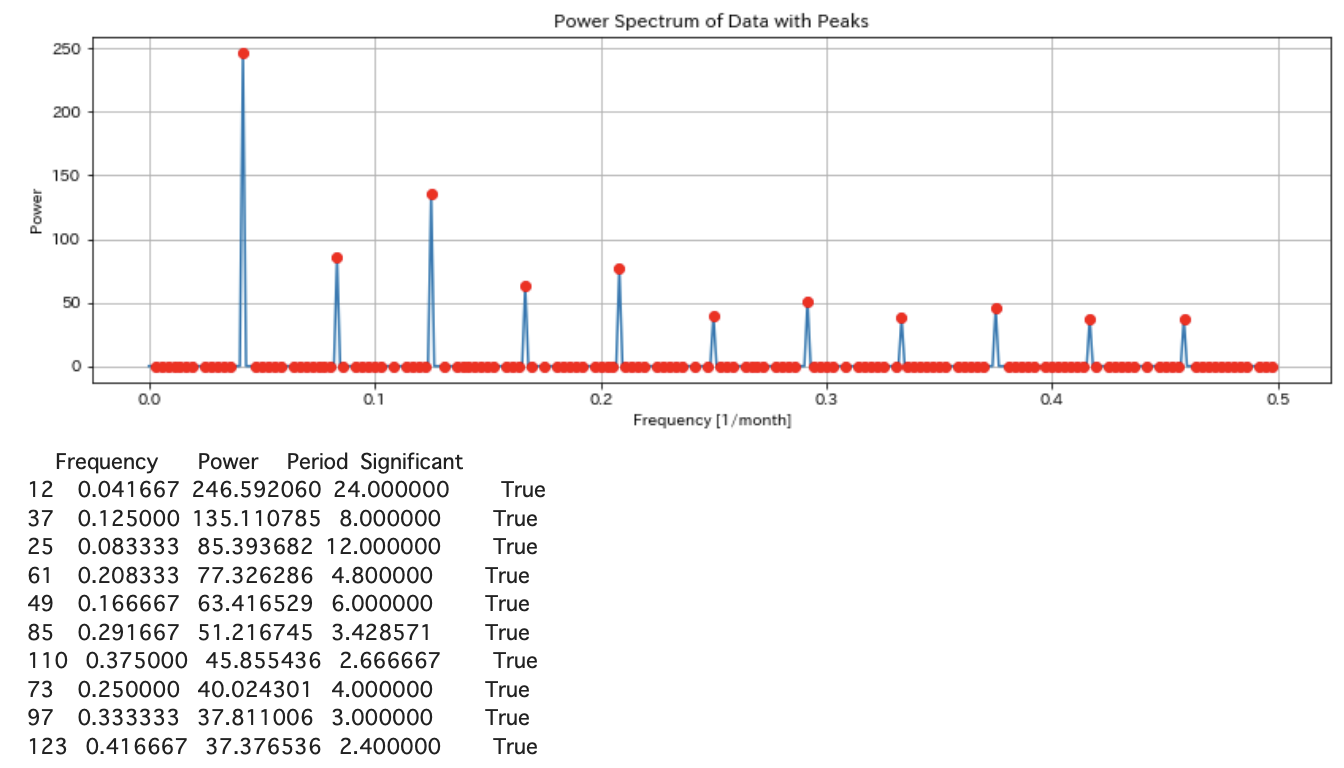
*

*Distance Walking Running*

*
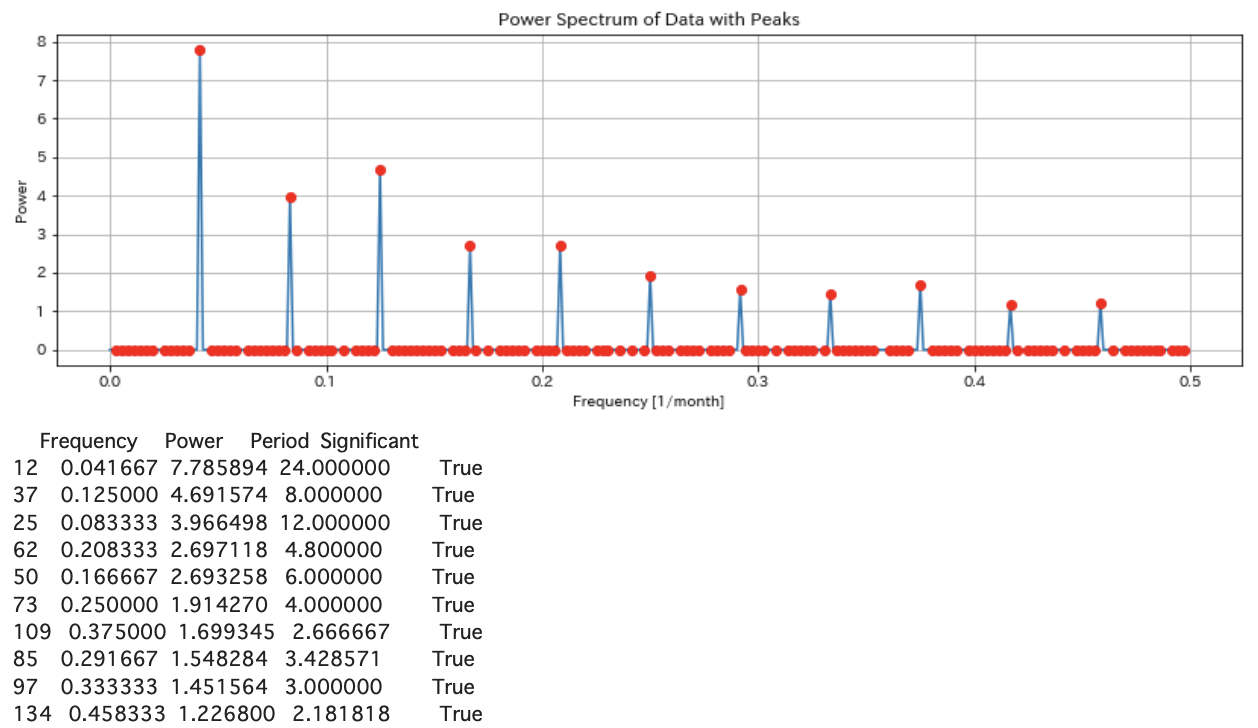
*

*Flights Climbed*

*
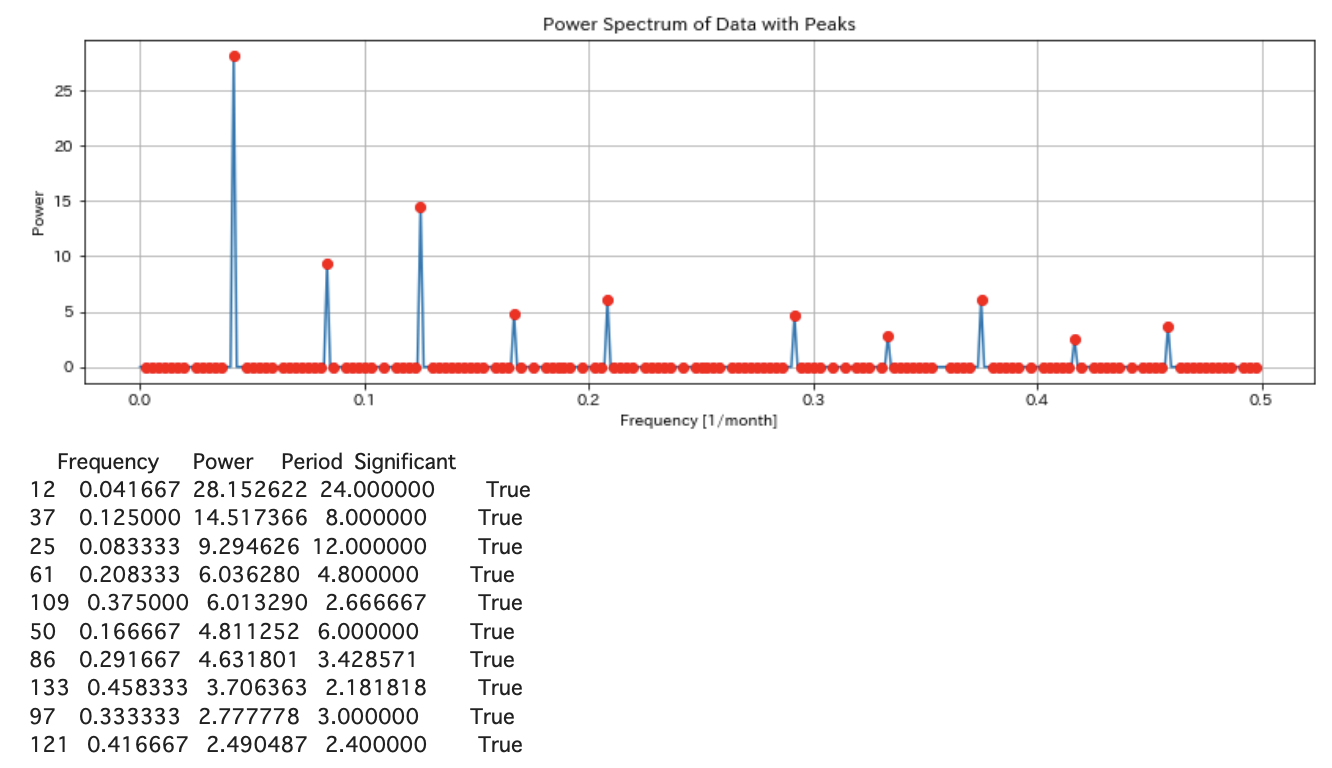
*

*Walking Step Length*


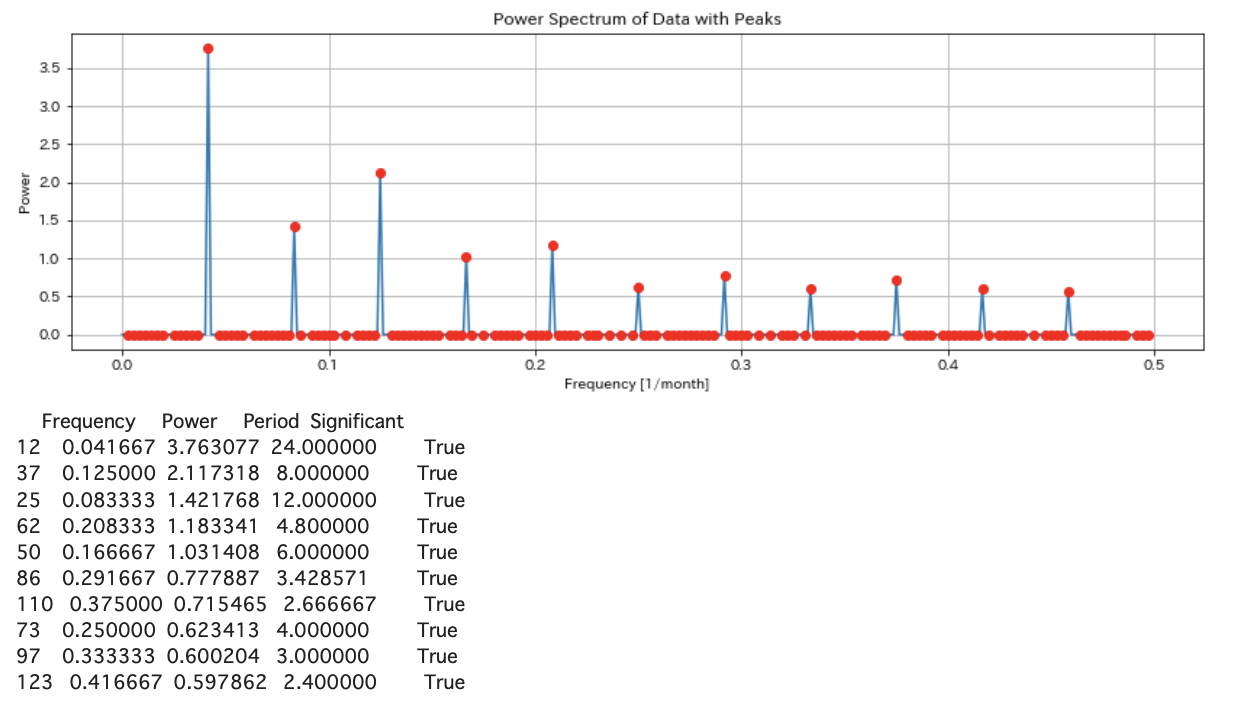


*Stand Time*

*
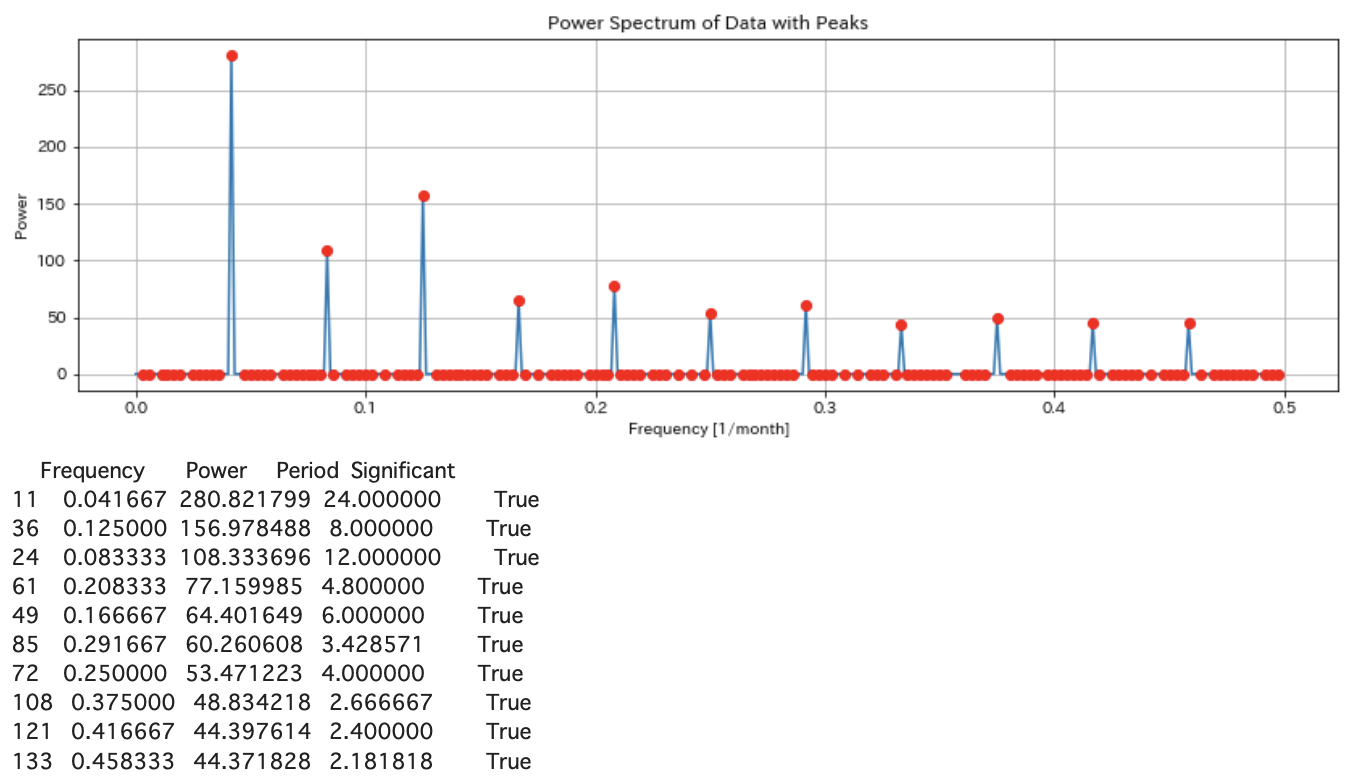
*

*Environmental Audio Exposure*

*
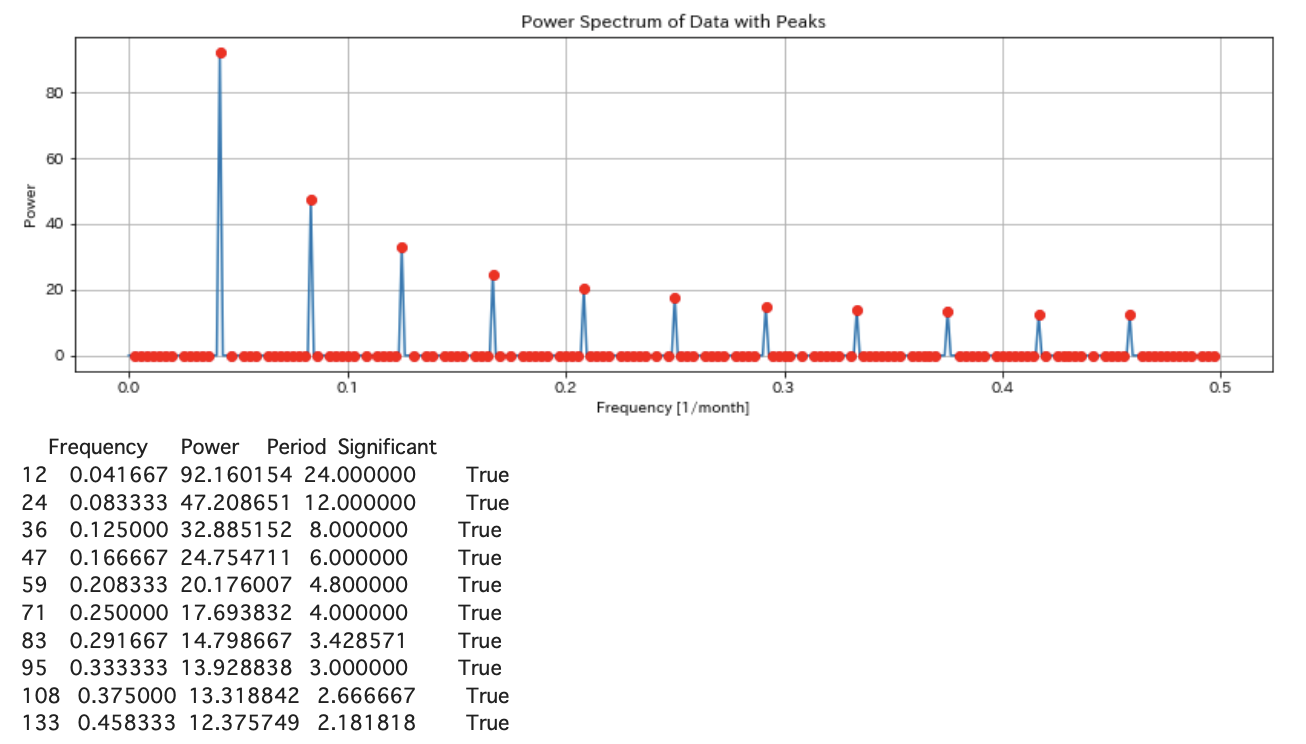
*

*Heart Rate*

*
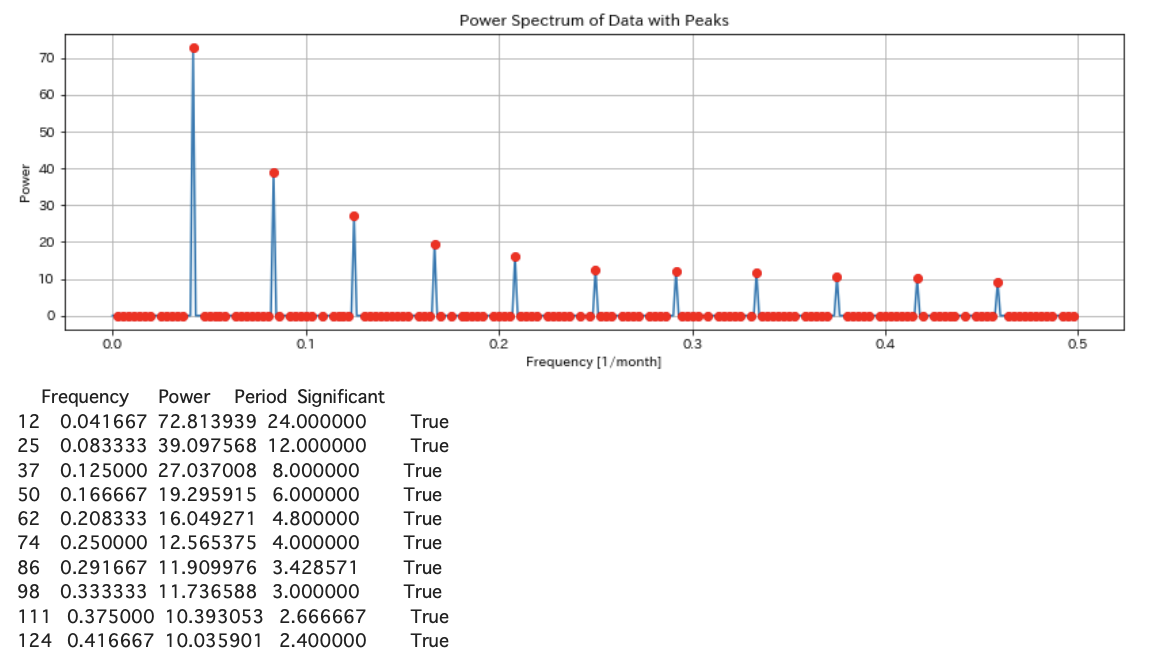
*
