# Supplementary material for: Construction of Personalized Predictive Models for Missed Medication Doses Using Wearable Device Data: Prospective Observational Study
Source: JMIR Form Res. 2025 Jun 24;9:e72113. doi: 10.2196/72113 (PMC12212888; doi:10.2196/72113)
Supplement: Multimedia Appendix 3 [file formative-v9-e72113-s003.docx]

Appendix 3. Individual Event Occurrence Rates and Dosing Regimen Details.

Table S1. Event Occurrence Rates per Number of Days for Each Participant.

| IDs | Total days | Number of events occurring | Event Occurrence Rates per Number of Days (%) |
| --- | --- | --- | --- |
| 1 | 30 | 24 | 20.0 |
| 2 | 30 | 17 | 43.3 |
| 3 | 29 | 13 | 58.6 |
| 4 | 30 | 4 | 86.7 |
| 5 | 29 | 1 | 96.6 |
| 6 | 30 | 0 | 100.0 |
| 7 | 30 | 0 | 100.0 |
| 8 | 30 | 0 | 100.0 |

Table S2. Event Occurrence Rates per Total Number of Doses for Each Participant.

| IDs | Expected total number of doses in this study | Number of events occurring | Event Occurrence Rates per Total Number of Doses |
| --- | --- | --- | --- |
| 1 | 90 | 32 | 64.44444444 |
| 2 | 30 | 17 | 43.33333333 |
| 3 | 87 | 15 | 82.75862069 |
| 4 | 60 | 4 | 93.33333333 |
| 5 | 58 | 1 | 98.27586207 |
| 6 | 60 | 0 | 100 |
| 7 | 90 | 0 | 100 |
| 8 | 30 | 0 | 100 |
