# Supplementary material for: Construction of Personalized Predictive Models for Missed Medication Doses Using Wearable Device Data: Prospective Observational Study
Source: JMIR Form Res. 2025 Jun 24;9:e72113. doi: 10.2196/72113 (PMC12212888; doi:10.2196/72113)
Supplement: Multimedia Appendix 4 [file formative-v9-e72113-s004.docx]

Appendix3

3-1. Feature importance (Group CV model)

3-1-1. ID1: Evening (after a meal /before bed)

3-1-2. ID2: Evening (after a meal)

3-1-3 ID3: Afternoon (after a meal)

3-2. Feature importance (non-Rolling feature model)

3-2-1. ID1: Evening (after a meal /before bed)

3-2-2. ID2: Evening (after a meal)

3-2-3. ID3: Afternoon (after a meal)
