# Supplementary material for: Construction of Personalized Predictive Models for Missed Medication Doses Using Wearable Device Data: Prospective Observational Study
Source: JMIR Form Res. 2025 Jun 24;9:e72113. doi: 10.2196/72113 (PMC12212888; doi:10.2196/72113)
Supplement: Checklist 1 [file formative-v9-e72113-s005.docx]

**Consolidated reporting guidelines for prognostic and diagnostic machine learning modeling studies**

**Author Checklist**

The following is the reporting checklist. A response should indicate whether the particular item is documented in the study. If the response to an item is Y then the location in the article should be provided (e.g., section number), and if the response is N or NA then some reasoning should be provided.

| **#** | **Item** | **Y** | **N** | **NA** | **Location / Reasoning** |
| --- | --- | --- | --- | --- | --- |
| **Study Details** | | | | | |
| 1.1 | *The medical/clinical task of interest* | ✓ |  |  | Introduction, Conclusion |
| 1.2 | *The research question* | ✓ |  |  | Introduction |
| 1.3 | *Current medical/clinical practice* | ✓ |  |  | Introduction |
| 1.4 | *The known predictors and confounders to what is being predicted / diagnosed* | ✓ |  |  | Introduction, Discussion (Factors Influencing Afternoon Model Performance, Feature Importance in the Two Models) |
| 1.5 | *The overall study design* | ✓ |  |  | Methods (Development of Data Collection Platform and Patient Recruitment) |
| 1.6 | *The medical institutional setting(s)* | ✓ |  |  | Methods (Development of Data Collection Platform and Patient Recruitment) |
| 1.7 | *The target patient population* | ✓ |  |  | Methods (Development of Data Collection Platform and Patient Recruitment) |
| 1.8 | *The intended use of the ML model* | ✓ |  |  | Introduction, Discussion (Findings, Broader Applicability) |
| 1.9 | *Existing model performance benchmarks for this task* |  | ✓ |  | We can’t find Existing model performance benchmarks. |
| 1.10 | *Ethical and other regulatory approvals obtained* | ✓ |  |  | Methods (Ethical Considerations) |
| **The Data** | | | | | |
| 2.1 | *Inclusion / exclusion criteria for the patient cohort* | ✓ |  |  | Methods (Development of Data Collection Platform and Patient Recruitment) |
| 2.2 | *Methods of data collection* | ✓ |  |  | Methods (Development of Data Collection Platform and Patient Recruitment, Preprocessing Before Model Construction and Verification of Time-Series Components) |
| 2.3 | *Bias introduced due to the method of data collection used* | ✓ |  |  | Methods (Development of Data Collection Platform and Patient Recruitment); Discussion (Limitations) |
| 2.4 | *Data characteristics* | ✓ |  |  | Methods (Development of Data Collection Platform and Patient Recruitment); Results (Aggregation of Event Occurrence Rates, Table 1) |
| 2.5 | *Methods of data transformations and preprocessing applied* | ✓ |  |  | Methods (Preprocessing Before Model Construction and Verification of Time-Series Components, Confirmation of Response Variables and Creation of Time-Series Features) |
| 2.6 | *Known quality issues with the data* | ✓ |  |  | Methods (Development of Data Collection Platform and Patient Recruitment); Discussion (Limitations) |
| 2.7 | *Sample size calculation* |  | ✓ |  | This study used a convenience sample of participants who completed the study. |
| 2.8 | *Data Availability* | ✓ |  |  | Data Availability section |
| **Methodology** | | | | | |
| 3.1 | *Strategies for handling missing data* | ✓ |  |  | Methods (Development of Data Collection Platform and Patient Recruitment) |
| 3.2 | *Strategies for addressing class imbalance* | ✓ |  |  | Methods (Confirmation of Response Variables and Creation of Time-Series Features, Construction of Predictive Models). Our approach ensures modeling is done on less severely imbalanced subsets. No explicit re-sampling techniques were used. |
| 3.3 | *Strategies for reducing dimensionality of data* |  | ✓ |  | This strategy was not considered necessary for the study methodology |
| 3.4 | *Strategies for handling outliers* | ✓ |  |  | Method. The data is subjected to statistical processing such as averaging within a certain range before being input into the model. |
| 3.5 | *Strategies for data augmentation* |  |  | ✓ | This strategy was not considered necessary for the study methodology |
| 3.6 | *Strategies for model pre-training* |  |  | ✓ | This strategy was not considered necessary for the study methodology |
| 3.7 | *The rationale for selecting the machine learning algorithm* | ✓ |  |  | Methods (Construction of Predictive Models) |
| 3.8 | *The method of evaluating model performance during training* | ✓ |  |  | Methods (Construction of Predictive Models) |
| 3.9 | *The method used for hyperparameter tuning* |  |  | ✓ | This strategy was not considered necessary for the study methodology |
| 3.10 | *Model’s output adjustments* |  | ✓ |  | The manuscript does not describe specific post-hoc adjustments to the model's output probabilities or predictions, beyond what is implicitly handled by optimizing for the F1 score. |
| **Evaluation** | | | | | |
| 4.1 | *Performance metrics used to evaluate the model* | ✓ |  |  | Methods (Construction of Predictive Models); Results (Predictive Models, Table 2, Table 3) |
| 4.2 | *The cost or consequence of errors* | ✓ |  |  | Discussion (High Precision and Low Recall in the Models) |
| 4.3 | *The results of internal validation* | ✓ |  |  | Results (Predictive Models); Results (Table 2, Table 3) |
| 4.4 | *The final model hyperparameters* | ✓ |  |  | Multimedia Appendix 2 |
| 4.5 | *Model evaluation on an external dataset* | ✓ |  |  | Discussion (Limitations) |
| 4.6 | *Characteristics relevant for detecting data shift and drift* | ✓ |  |  | Discussion (Limitations) |
| **Explainability and Transparency** | | | | | |
| 5.1 | *The most important features and how they relate to the outcome(s)* | ✓ |  |  | Results (Feature Importance); Discussion (Feature Importance in the Two Models) |
| 5.2 | *Plausibility of model outputs* | ✓ |  |  | Results (Predictive Models); Discussion |
| 5.3 | *Interpretation of model's results by an end-user* |  | ✓ |  | The current study focuses on the development and internal validation of the predictive models. |
